# Genomic Features Associated with the Degree of Phenotypic Resistance to Carbapenems in Carbapenem-Resistant Klebsiella pneumoniae

**DOI:** 10.1128/mSystems.00194-21

**Published:** 2021-09-14

**Authors:** Zackery P. Bulman, Fiorella Krapp, Nathan B. Pincus, Eric Wenzler, Katherine R. Murphy, Chao Qi, Egon A. Ozer, Alan R. Hauser

**Affiliations:** a Department of Pharmacy Practice, University of Illinois at Chicago, Chicago, Illinois, USA; b Department of Medicine, Division of Infectious Diseases, Northwestern University Feinberg School of Medicine, Chicago, Illinois, USA; c Department of Microbiology-Immunology, Northwestern University Feinberg School of Medicine, Chicago, Illinois, USA; d Department of Pathology, Northwestern University Feinberg School of Medicine, Chicago, Illinois, USA; Marquette University

**Keywords:** carbapenem, *Klebsiella pneumoniae*, whole-genome sequencing, machine learning, antibiotic resistance

## Abstract

Carbapenem-resistant Klebsiella pneumoniae strains cause severe infections that are difficult to treat. The production of carbapenemases such as the K. pneumoniae carbapenemase (KPC) is a common mechanism by which these strains resist killing by the carbapenems. However, the degree of phenotypic carbapenem resistance (MIC) may differ markedly between isolates with similar carbapenemase genes, suggesting that our understanding of the underlying mechanisms of carbapenem resistance remains incomplete. To address this problem, we determined the whole-genome sequences of 166 K. pneumoniae clinical isolates resistant to meropenem, imipenem, or ertapenem. Multiple linear regression analysis of this collection of largely *bla*_KPC-3_-containing sequence type 258 (ST258) isolates indicated that *bla*_KPC_ copy number and some outer membrane porin gene mutations were associated with higher MICs to carbapenems. A trend toward higher MICs was also observed with those *bla*_KPC_ genes carried by the *d* isoform of Tn*4401*. In contrast, *ompK37* mutations were associated with lower carbapenem MICs, and extended spectrum β-lactamase genes were not associated with higher or lower MICs in carbapenem-resistant K. pneumoniae. A machine learning approach based on the whole-genome sequences of these isolates did not result in a substantial improvement in prediction of isolates with high or low MICs. These results build upon previous findings suggesting that multiple factors influence the overall carbapenem resistance levels in carbapenem-resistant K. pneumoniae isolates.

**IMPORTANCE** Klebsiella pneumoniae can cause severe infections in the blood, urinary tract, and lungs. Resistance to carbapenems in K. pneumoniae is an urgent public health threat, since it can make these isolates difficult to treat. While individual contributors to carbapenem resistance in K. pneumoniae have been studied, few reports explore their combined effects in clinical isolates. We sequenced 166 clinical carbapenem-resistant K. pneumoniae isolates to evaluate the contribution of known genes to carbapenem MICs and to try to identify novel genes associated with higher carbapenem MICs. The *bla*_KPC_ copy number and some outer membrane porin gene mutations were associated with higher carbapenem MICs. In contrast, mutations in one specific porin, *ompK37*, were associated with lower carbapenem MICs. Machine learning did not result in a substantial improvement in the prediction of carbapenem resistance nor did it identify novel genes associated with carbapenem resistance. These findings enhance our understanding of the many contributors to carbapenem resistance in K. pneumoniae.

## INTRODUCTION

Carbapenem-resistant *Enterobacterales* (CRE) are urgent public health threats and critical priority pathogens as defined by the World Health Organization (1). CRE are considered a leading threat in part due to their global spread and the limited availability of treatment options. Furthermore, infections caused by CRE are associated with high mortality rates, between ∼20 and 50% (2–4). The most common *Enterobacterales* species that are resistant to carbapenems include Klebsiella pneumoniae, Enterobacter spp., and Escherichia coli. Among these species, K. pneumoniae has been found to account for 57% to 74% of all CRE infections (2, 5) and 85% to 90% of bloodstream infections caused by CRE (3, 4). Carbapenem resistance can be caused by a variety of different mechanisms, and the prevalence of each varies by geographic region.

Production of a carbapenemase is the most commonly reported mechanism of carbapenem resistance among K. pneumoniae isolates worldwide. Molecular diagnostic approaches have taken advantage of the fact that a vast majority of carbapenem-resistant Klebsiella pneumoniae (CR-Kp) isolates harbor a carbapenemase gene such as the K. pneumoniae carbapenemase (*bla*_KPC_) or New-Delhi-metallo-β-lactamase (*bla*_NDM_) genes. The presence of a carbapenemase gene usually predicts phenotypic carbapenem resistance, though the degree of carbapenem resistance (MIC) among these isolates greatly varies (6). This variability in the carbapenem MIC is likely driven by the presence of other genotypic factors, which also contribute to carbapenem resistance in both carbapenemase- and non-carbapenemase-producing CRE. Some genotypic variations observed in CR-Kp, such as changes in carbapenemase gene copy number (7–10) or variations in transposon isoform and promoter regions (11, 12), modify the expression level of carbapenemase genes and may explain some of the variability in carbapenem MICs. Other factors not associated with carbapenemase expression that may also increase carbapenem MICs include the expression of extended spectrum β-lactamases (ESBLs), which are especially problematic in conjunction with decreased carbapenem membrane permeability related to porin deficits (13, 14). These contributors to carbapenem resistance have largely been investigated in isolation, and few data exist to validate their combined influence on carbapenem MIC in clinical CR-Kp collections. We performed genomic characterization of 166 clinical K. pneumoniae isolates to evaluate genetic correlates with levels of carbapenem resistance.

## RESULTS

### CR-Kp isolate characteristics.

A total of 174 K. pneumoniae clinical isolates recovered from patients at Northwestern Memorial Hospital between October 2010 and February 2016 were identified as being resistant to at least one carbapenem by the clinical microbiology laboratory (Vitek 2). To confirm resistance, Etests were performed on each isolate. Eight of these isolates were excluded because Etest results did not confirm resistance to any of the carbapenems; thus, 166 CR-Kp isolates were included in the final analyses (see Table S1 in the supplemental material). Within this collection, rates of resistance to ertapenem, imipenem, and meropenem as defined by Etest were 97.6% (162/166), 77.1% (128/166), and 71.1% (118/166), respectively. Collection rates of CR-Kp isolates did not appreciably change between study onset and conclusion, with ∼2 to 3 isolates collected per month throughout each year of the study (see Fig. S1). Urine was the most common source of the isolates (*n* = 87), followed by the respiratory tract (*n* = 46), blood (*n* = 13), and other miscellaneous sources (*n* = 20) (Table S1).

10.1128/mSystems.00194-21.1 TABLE S1 Characteristics of the 166 CR-K. pneumoniae isolates examined in this study. Download Table S1, PDF file, 0.1 MB. Copyright © 2021 Bulman et al. 2021 Bulman et al. https://creativecommons.org/licenses/by/4.0/ This content is distributed under the terms of the Creative Commons Attribution 4.0 International license.

10.1128/mSystems.00194-21.2 FIG S1 Mean number of CR-Kp isolates collected each month during the study period from 2010 to 2016. Mean was calculated by dividing the total number of CR-Kp isolates obtained each year by the number of months the study was ongoing in the given year. Download FIG S1, PDF file, 0.04 MB. Copyright © 2021 Bulman et al. 2021 Bulman et al. https://creativecommons.org/licenses/by/4.0/ This content is distributed under the terms of the Creative Commons Attribution 4.0 International license.

**FIG 1:**
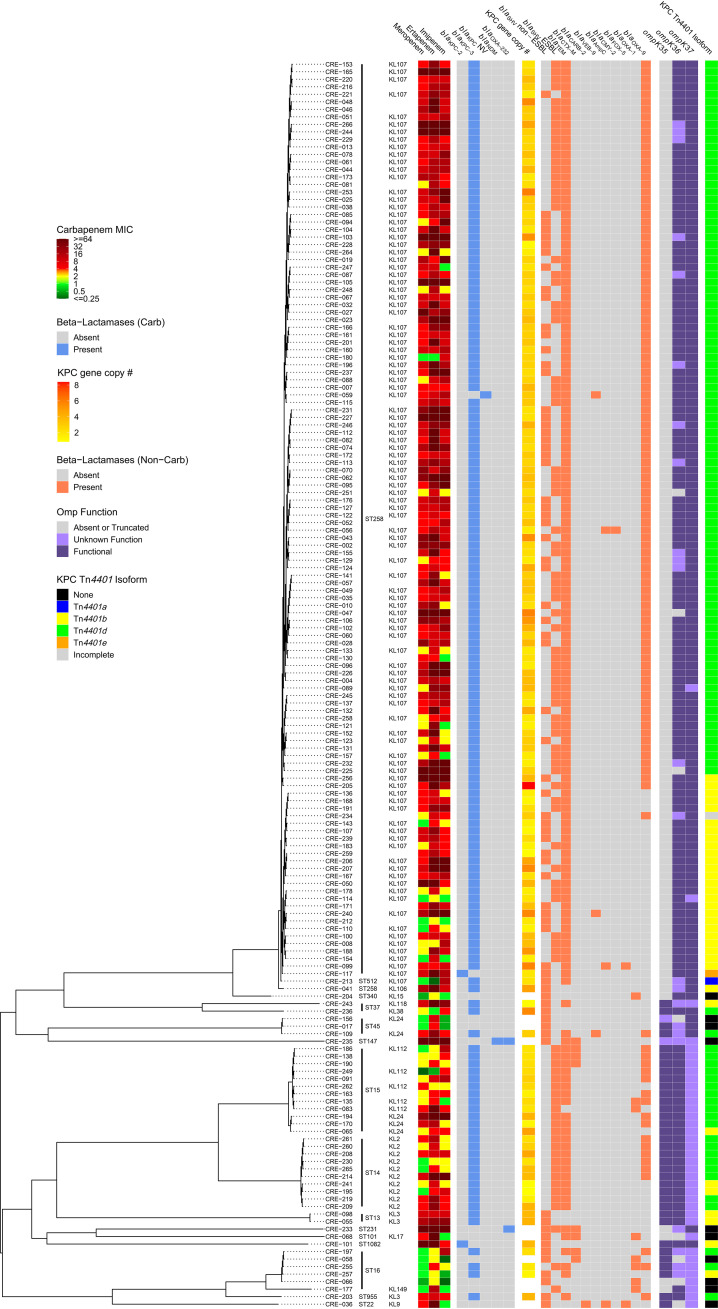
Core genome phylogenetic tree of the 166 CR-Kp isolates included in the final analyses and corresponding genotypic/phenotypic isolate information. Sequence type and capsule locus type (if predicted) are listed next to each isolate. Carbapenem MICs to meropenem, ertapenem, and imipenem are indicated by the gradient from green (low MIC) to red (high MIC). The presence of carbapenemase or other β-lactamase genes is depicted by blue and orange boxes, respectively. The *bla*_KPC_ copy number is presented with a gradient from yellow (low) to red (high). The status of the *ompK35*, *ompK36*, and *ompK37* outer membrane porin channel genes are designated by the dark purple (wild-type allele), light purple (allele with mutations of unclear significance), and gray (absent or truncated allele) boxes. Transposon isoforms are indicated in blue (Tn*4401a*), yellow (Tn*4401b*), green (Tn*44401d*), and orange (Tn*4401e*). The ertapenem breakpoints for susceptibility and resistance are ≤0.5 mg/liter and ≥2 mg/liter, respectively. The imipenem and meropenem breakpoints for susceptibility and resistance are ≤1 mg/liter and ≥4 mg/liter, respectively.

**FIG 2:**
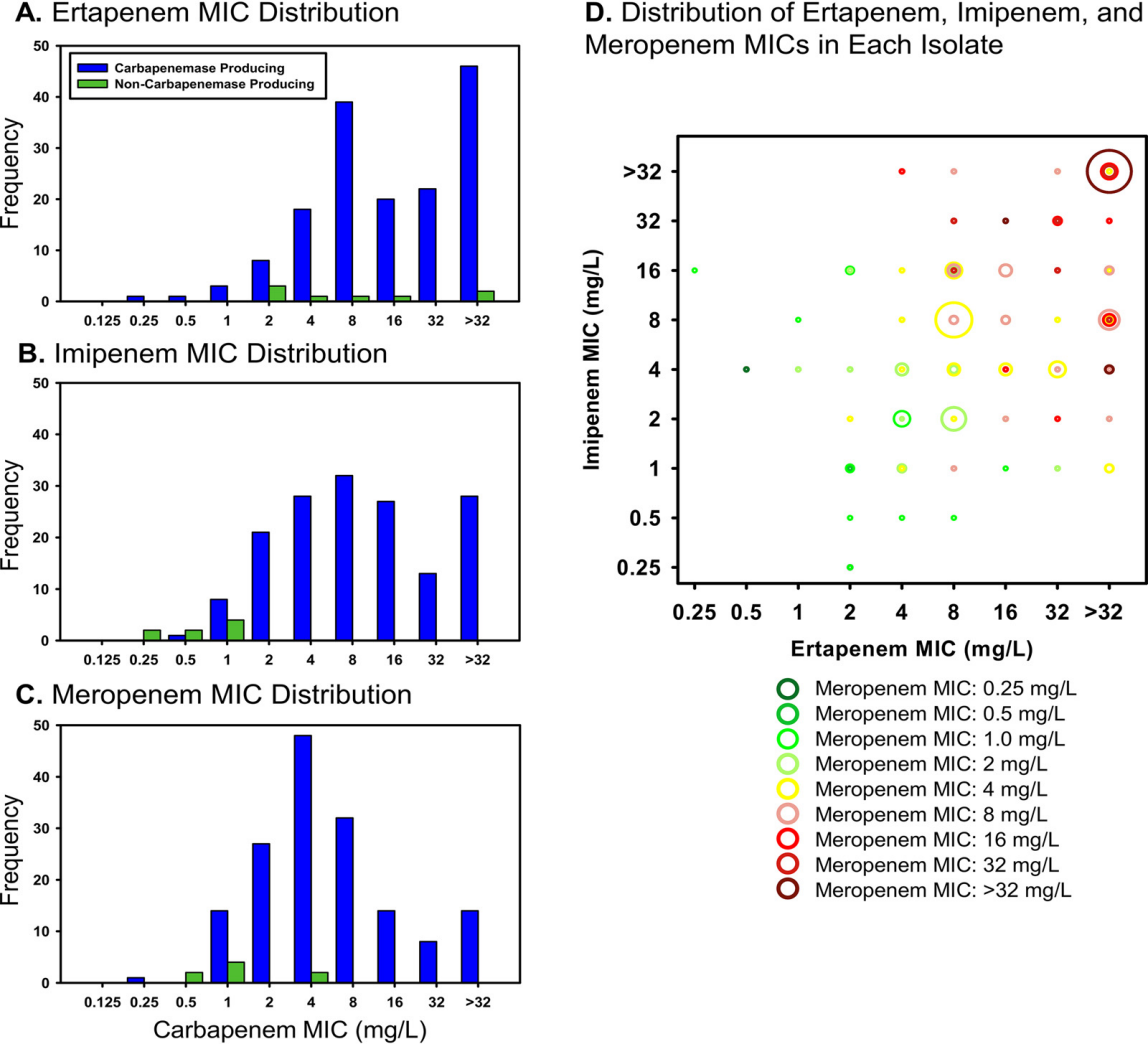
Distribution of MICs obtained from Etest for ertapenem (A), imipenem (B), and meropenem (C) for CR-Kp isolates that contain carbapenemases (blue) and those without carbapenemases (green). (D) The distribution of phenotypic MICs of all three carbapenems in each CR-Kp isolate. Circles represent isolates with the corresponding ertapenem, imipenem, and meropenem MICs; the color of the circle corresponds to the meropenem MIC, and the size of the circle corresponds to the number of isolates with that phenotype (e.g., the large brown circle in the upper right corner represents 11 CR-Kp isolates that have ertapenem, imipenem, and meropenem MICs of >32, >32, and >32 mg/liter, respectively).

Short-read whole-genome sequencing was performed on each isolate. A majority of the CR-Kp isolates (74.1% [123/166]) belonged to sequence type 258 (ST258), while there were 10, 12, and 6 isolates in ST14, ST15, and ST16, respectively (Fig. 1). A majority of the CR-Kp isolates had the capsule locus type KL107 (62.7% [104/166]), while the next most common were categorized as KL2 (6.0% [10/166]), KL24 (3.0% [5/166]), KL112 (3.0% [5/166]), or KL3 (1.8% [3/166]). Of the remaining CR-Kp, 32 (19.3%) did not have a defined KL type, and 7 isolates were categorized into 7 different KL types (Table S1). Examination of the sequences revealed that 158 (95.2%) of the 166 CR-Kp isolates carried at least one carbapenemase gene (Fig. 1). On average, the non-carbapenemase-harboring CR-Kp (*n* = 8) had imipenem and meropenem MICs that were lower than those for the isolates that contained carbapenemases, while the ertapenem MIC distributions were similar for both isolates with and without a carbapenemase gene (Fig. 2A to C). Considering the intraisolate phenotype, MICs tended to increase for all three carbapenems together (Fig. 2D). However, there were a few isolates that had a disproportionately elevated ertapenem MIC relative to the imipenem and meropenem MICs. The *bla*_KPC-3_ gene was the most common carbapenemase gene and was present in 153 isolates (92.2% of total isolates), while the *bla*_KPC-2_ gene was detected in 2 isolates (1.2% of total isolates). Of the remaining 3 CR-Kp isolates harboring a carbapenemase gene, 1 contained *bla*_OXA-232_, 1 contained both *bla*_OXA-232_ and *bla*_NDM-7_, and 1 contained a novel *bla*_KPC_ variant. This variant differed by a single nucleotide (G436A) from *bla*_KPC-3_, which resulted in the substitution of an arginine for a glycine residue at position 146 in the KPC-3 protein (GenBank AEV55249.1 numbering system). This substitution lies in an alpha-helix outside the active site. In addition, each isolate carried between 2 and 5 genes coding for other β-lactamases of limited or extended spectrum.

Our findings indicated that carbapenem resistance in K. pneumoniae isolates from our collection site was largely due to *bla*_KPC-3_. However, despite carrying the same carbapenemase gene, these *bla*_KPC-3_-positive isolates differed markedly in their MICs. For each isolate, it was verified that the *bla*_KPC-3_ gene sequence was identical. Since the carbapenem MIC could not be predicted based on the presence of a carbapenemase gene alone, additional genotypic-phenotypic relationships were explored.

### Associations between genotypes and carbapenem resistance.

**FIG 3:**
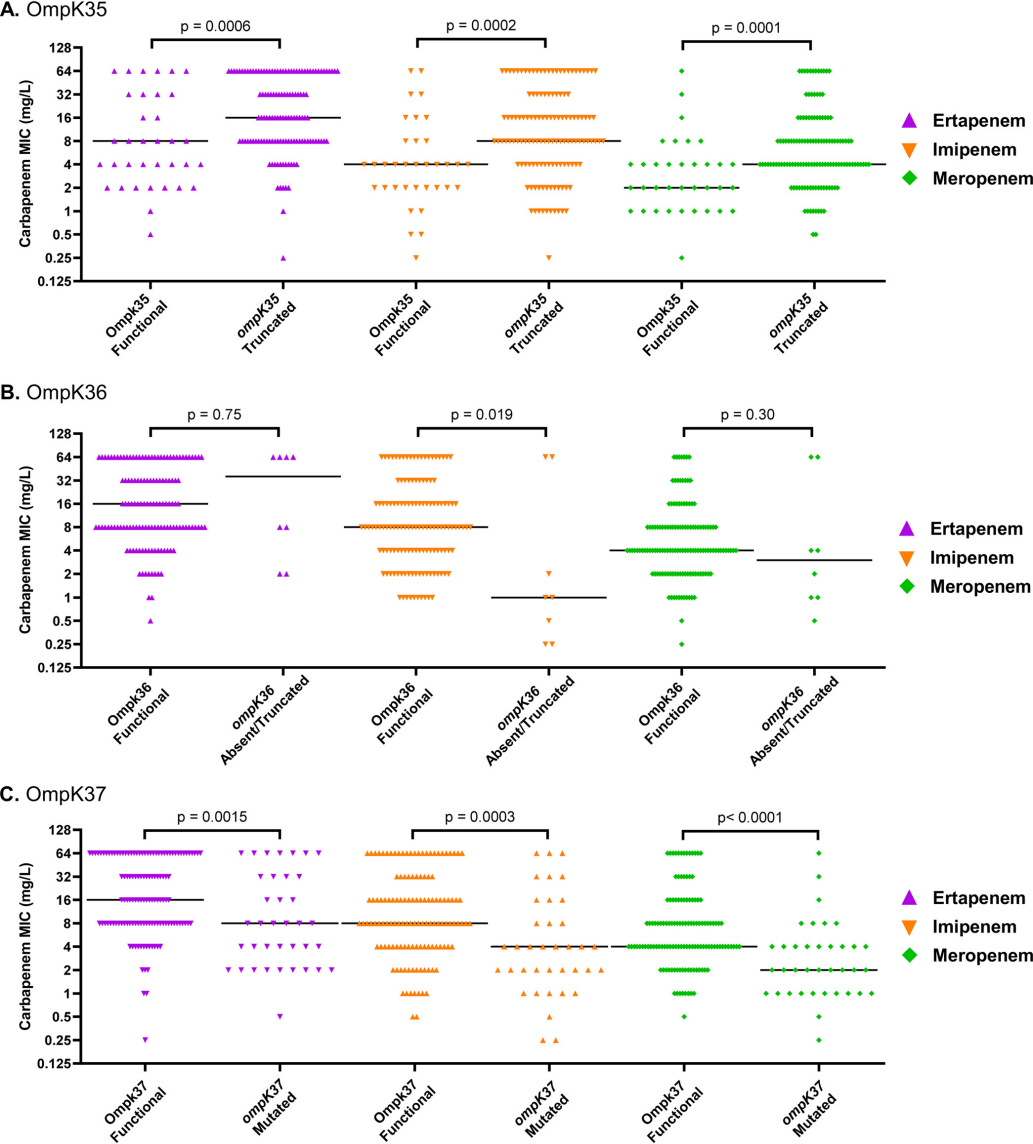
Ertapenem (purple), imipenem (orange), and meropenem (green) MICs for each CR-Kp isolate stratified by the predicted function of OmpK35 (A), OmpK36 (B), and OmpK37 (C) outer membrane proteins. Black lines represent the median MIC for each group. MIC values of >32 mg/liter were converted to 64 mg/liter for analysis.

**(i) Outer membrane porin channel alterations.** Outer membrane porins allow for the passage of β-lactams, including carbapenems, to their binding site in the bacterial periplasmic space. Therefore, we evaluated the association between the *ompK35*, *ompK36*, and *ompK37* genotypes and the carbapenem MICs. For *ompK35*, 35 (21.1%) CR-Kp isolates were predicted to have functional OmpK35 porin channels, whereas 129 (77.7%) had a premature stop codon or insertion sequence in the *ompK35* gene (two isolates could not definitively be associated with a phenotype and were excluded from these analyses). Presence of a premature stop codon or insertion sequence in the *ompK35* gene was associated with a 1-log_2_ dilution median increase in the ertapenem, imipenem, and meropenem MICs (*P* < 0.005) (Fig. 3A). Conversely, the *ompK36* genes for a majority of the CR-Kp isolates (135; 81.3%) were identical to wild-type genes and predicted to encode functional OmpK36 proteins, while only 8 isolates (4.8%) had a premature stop codon or were missing the *ompK36* gene. Among the 8 isolates with an absent or truncated *ompK36*, imipenem MICs were significantly lower (*P* = 0.019) (Fig. 3B). However, no significant change in the ertapenem or meropenem MIC was observed (*P* > 0.05). The remaining 23 CR-Kp isolates had mutations in the *ompK36* gene, but function of the porin channel could not be predicted, which excluded them from these porin channel analyses. There were only 5 (3.0%) CR-Kp isolates with both absent or truncated *ompK35* and *ompK36* genes, and their median MICs for ertapenem, imipenem, and meropenem were 16, 8, and 4 mg/liter, respectively. Lastly, none of the isolates harbored *ompK37* genes with premature stop codons, though 37 (22.3%) contained a mutation that did not have a previously defined effect on the function of OmpK37 porin channels. Interestingly, the isolates with *ompK37* mutations had ertapenem, imipenem, and meropenem median MICs that were 2-fold less than that for the CR-Kp with wild-type *ompK37* genes, which was a significant decrease (*P* < 0.005 for each carbapenem) (Fig. 3C).

**(ii) Presence of ESBL genes.** Although noncarbapenemase β-lactamases such as ESBLs have little capacity to hydrolyze carbapenems, previous data have shown they may contribute to carbapenem resistance in some isolates (15). The relationship between the presence of ESBL genes and carbapenem MICs in the CR-Kp isolates was explored. CR-Kp isolates with (*n* = 100; 60.2%) and without (*n* = 66; 39.8%) an ESBL gene did not have significantly different MICs for any of the carbapenems (*P* > 0.05) (see Fig. S2). The subset of CR-Kp isolates with both an ESBL gene and a truncated *ompK35* allele had significantly higher carbapenem MICs (*P* < 0.005) than isolates with an ESBL gene and predicted functional OmpK35; however, this appeared to be driven by the nonfunctional porin channel, not the cocarriage of the ESBL (see Fig. S3A). Among isolates with a truncated *ompK35* porin gene, the cocarriage of an ESBL did not increase the median carbapenem MICs, and mean carbapenem MICs only increased between 1.43 and 6.72 mg/liter. CR-Kp isolates with an ESBL gene and an absent or truncated *ompK36* gene did not have carbapenem MICs that significantly different from isolates with a wild-type *ompK36* gene (*P* > 0.05) or without an ESBL gene (*P* > 0.05), and no trend was noted, though there were only 5 CR-Kp isolates with this genotype (3.0%) (Fig. S3B). Thus, ESBLs may play at most a minor role to increase carbapenem MICs within CR-Kp isolates that coharbor carbapenemase genes.

10.1128/mSystems.00194-21.3 FIG S2 Ertapenem (purple), imipenem (orange), and meropenem (green) MICs for each CR-Kp isolate stratified by the presence of an ESBL gene. MIC values of >32 mg/liter were converted to 64 mg/liter for analysis. Download FIG S2, PDF file, 0.07 MB. Copyright © 2021 Bulman et al. 2021 Bulman et al. https://creativecommons.org/licenses/by/4.0/ This content is distributed under the terms of the Creative Commons Attribution 4.0 International license.

10.1128/mSystems.00194-21.4 FIG S3 (A) Ertapenem (purple), imipenem (orange), and meropenem (green) MICs for the subsets of CR-Kp isolates with or without an ESBL stratified by the predicted function of OmpK35. (B) Ertapenem (purple), imipenem (orange), and meropenem (green) MICs for the subsets of CR-Kp isolates with or without an ESBL stratified by the predicted function of OmpK36. Black lines represent the median MIC for each group. MIC values of >32 mg/liter were converted to 64 mg/liter for analysis. Download FIG S3, PDF file, 0.2 MB. Copyright © 2021 Bulman et al. 2021 Bulman et al. https://creativecommons.org/licenses/by/4.0/ This content is distributed under the terms of the Creative Commons Attribution 4.0 International license.

**FIG 4:**
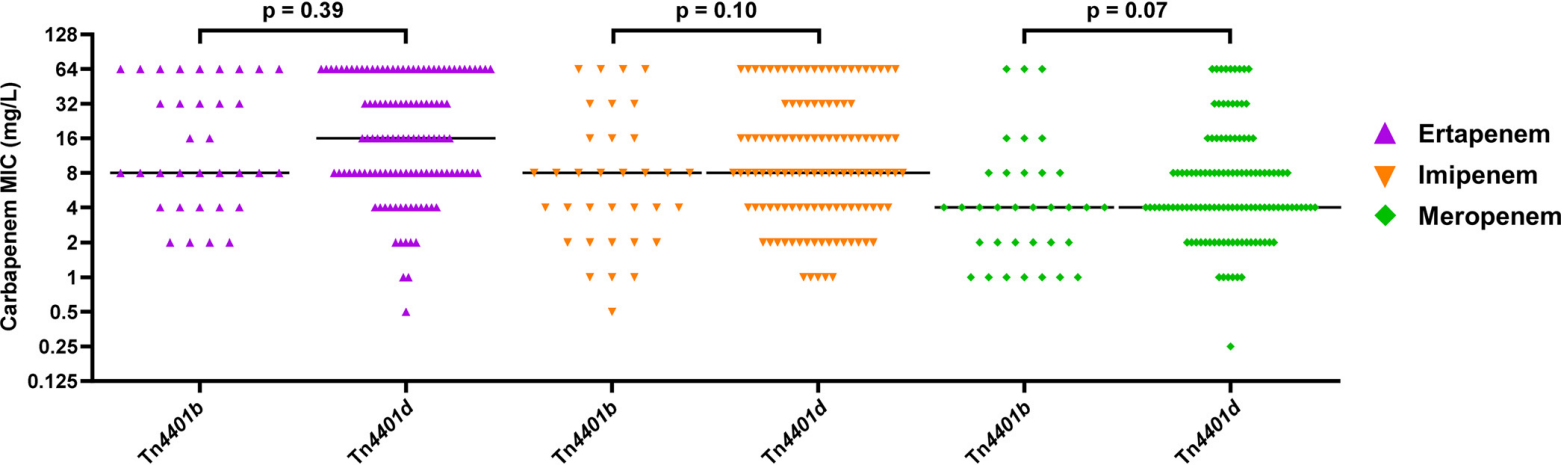
Ertapenem (purple), imipenem (orange), and meropenem (green) MICs for each CR-Kp isolate stratified by the type of transposon on which the *bla*_KPC_ gene was located. Black lines represent the median MIC for each group. MIC values of >32 mg/liter were converted to 64 mg/liter for analysis.

**(iii) Transposon harboring the *bla*_KPC_ gene.** Different Tn*4401* transposon isoforms that may affect *bla*_KPC_ expression are found in some CR-Kps (11, 12). Of the 156 CR-Kp isolates with *bla*_KPC_, a majority carried the *bla*_KPC_ gene in the Tn*4401d* transposon isoform (*n* = 119; 76.3%); one of these had one single nucleotide variation (SNV) (C6941A), while the others were identical to the Tn*4401d* reference sequence (7, 12). Next most common was the Tn*4401b* transposon isoform (*n* = 34; 21.8%), though only 3 isolates had sequences identical to the reference (accession CP017937); 1 isolate had 1 SNV in the P2 promoter (G6909A), while 30 isolates had 5 SNVs, 4 of which were in the P1 promoter region (T7148G, T7152G, A7153T, C7154A, and C7158A). There was also 1 isolate with the Tn*4401a* transposon, 1 isolate with the Tn*4401e* transposon, and 1 isolate in which the transposon fell at the end of a contig and could not be typed. The isolates that contained Tn*4401d* had carbapenem MICs that were on average 1.9 to 5.2 mg/liter higher than isolates with Tn*4401b* (median MICs of 0 or 1-log_2_ dilution difference), which was trending toward being a significant increase for imipenem and meropenem. (Fig. 4).

**FIG 5:**
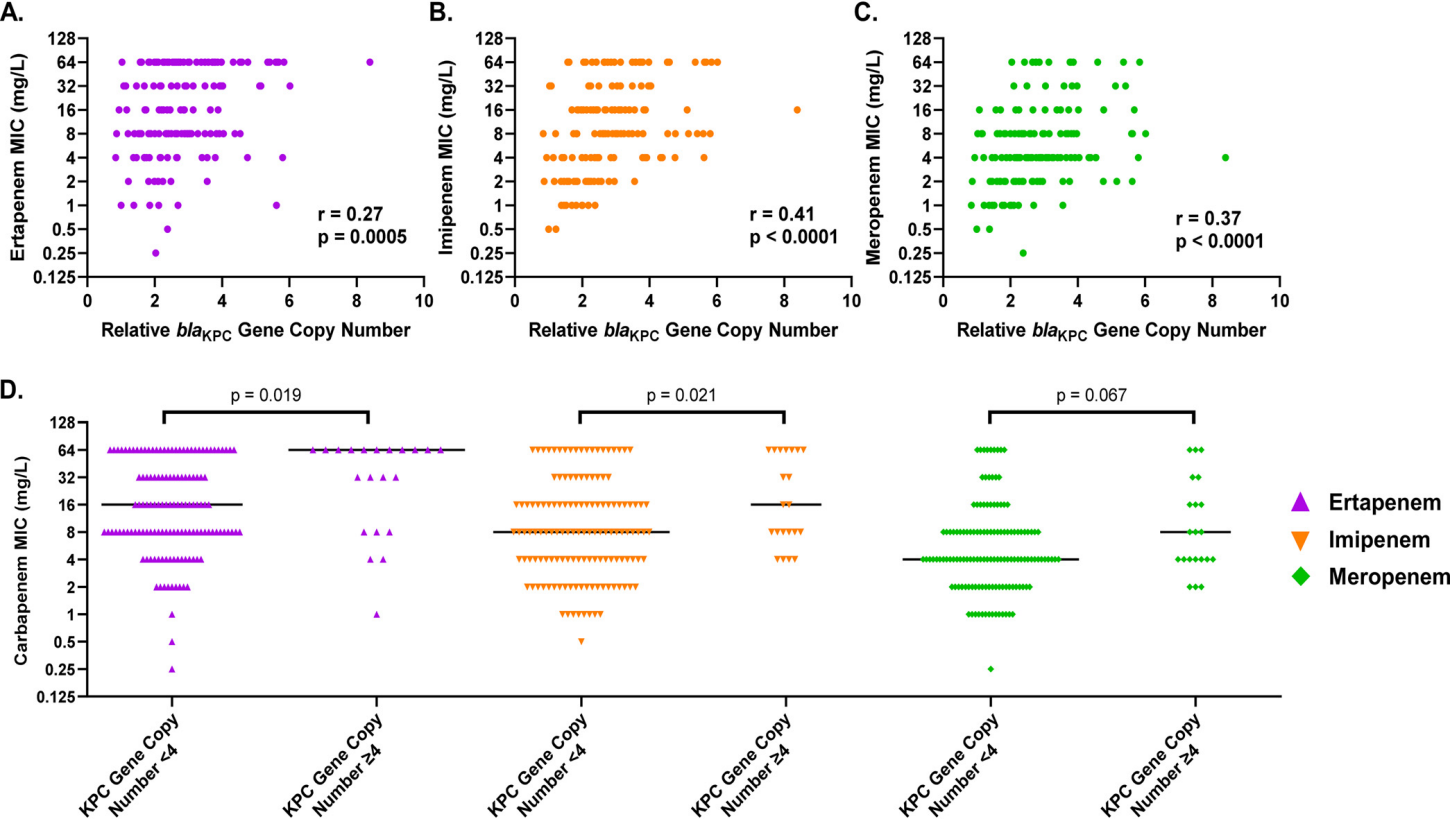
Relationship between the relative *bla*_KPC_ copy number in each CR-Kp isolate and its ertapenem (A), imipenem (B), and meropenem (C) MIC, where “r” represents the Spearman’s correlation coefficient. (D) Comparison of carbapenem MICs for isolates with fewer than 4 copies of the *bla*_KPC_ gene and those with ≥4 copies of the *bla*_KPC_ gene. Black lines represent the median MIC for each group. MIC values of >32 mg/liter were converted to 64 mg/liter for analysis.

**(iv) Relative copy number of the *bla*_KPC_ gene.** Higher *bla*_KPC_ copy number can lead to increased expression of the KPC enzyme and is associated with elevated carbapenem MICs (7). In the subgroup of CR-Kp isolates that harbored a *bla*_KPC_ gene (*bla*_KPC-2_ or *bla*_KPC-3_), a higher *bla*_KPC_ relative copy number was significantly correlated with higher MICs for ertapenem, imipenem, and meropenem (*P* < 0.005) (Fig. 5A to C). Though significant, the positive correlation was weak to moderate. The correlation was strongest for imipenem (*r* = 0.41), followed by meropenem (*r* = 0.37), and was the weakest for ertapenem MICs (*r* = 0.27). Compared to that for CR-Kp isolates estimated to have <4 copies of the *bla*_KPC_ gene (*n* = 135; 86.5%), isolates with ≥4 copies (*n* = 21; 13.5%) had 4-fold higher ertapenem (*P* = 0.019) and 2-fold higher imipenem median MICs (*P* = 0.021), while the meropenem median MIC was trending toward being significantly higher (*P* = 0.067) (Fig. 5D). These findings demonstrate that isolates with increased copy numbers of the *bla*_KPC_ gene tended to have elevated carbapenem MICs. In contrast to the correlation between MIC and the *bla*_KPC_ copy number, there was less of a correlation between the *bla*_ESBL_ copy number and the ertapenem (*r* = 0.24) or imipenem (*r* = 0.23) MIC; the correlation between *bla*_ESBL_ copy number and the meropenem MIC (*r* = 0.38) was similar to the correlation between meropenem MIC and *bla*_KPC_ copy number (data not shown).

The increased copy numbers of *bla*_KPC_ in some isolates could be the result of one of two mechanisms: duplication of the *bla*_KPC_ gene itself, which has been reported (16, 17), or increased copy number of the entire plasmid carrying *bla*_KPC_ (9). In nearly every isolate, the coverage ratio of the *bla*_KPC_ gene to the other 5 genes was between 80% and 120%, with most between 90% and 110%. From this we conclude that the increased *bla*_KPC_ copy number observed in most of the carbapenemase-producing isolates was likely due to increased copy number of the plasmid carrying *bla*_KPC_.

**(v) Multiple linear regression analysis.** Multiple linear regression analysis identified CR-Kp genes that were associated with significant changes in the MIC for each carbapenem evaluated. Final models all incorporated some established contributors to carbapenem resistance, including *bla*_KPC_ copy number, *bla*_OXA-232_ presence, and *ompK35* or *ompK36* alleles (Table 1). While the copy number of the ESBL genes was not associated with carbapenem MICs, each additional copy of the *bla*_KPC_ gene was associated with a carbapenem MIC increase of 3.37 to 4.85 mg/liter. The presence of *bla*_OXA-232_ was associated with MIC increases of 44.36 to 65.15 mg/liter. The meropenem model also showed that the presence of the wild-type *ompK37* gene was associated with significant increases in meropenem MIC (7.45 mg/liter). In general, models were only able to explain a small proportion of the carbapenem MIC variability among CR-Kp isolates (adjusted R^2^ <0.3).

**TABLE 1:** Multiple linear regression analysis demonstrating the influence of detected genotypic predictors on the MICs of ertapenem, imipenem, and meropenem against 166 CR-Kp isolates

Carbapenem	Molecular predictor	β	95% confidence interval	*P* value
Ertapenem^*a*^	*bla*_KPC_ copy no.	4.81	2.08 to 7.54	0.001
	*bla*_OXA-232_	44.36	9.85 to 78.87	0.12
	Truncated *ompK35*	10.07	1.28 to 18.86	0.025
	Wild-type *ompK36*	−10.63	−20.26 to −1.00	0.031
Imipenem^*b*^	*bla*_KPC_ copy no.	4.85	2.52–7.18	<0.001
	*bla*_OXA-232_	57.43	28.17 to 86.70	<0.001
	Wild-type *ompK35*	−9.09	−16.76 to −1.42	0.021
Meropenem^*c*^	*bla*_KPC_ copy no.	3.37	1.52 to 5.21	<0.001
	*bla*_OXA-232_	65.15	42.75 to 87.54	<0.001
	Absent or truncated *ompK36*	14.10	2.63 to 25.58	0.016
	Wild-type *ompK37*	7.45	1.56 to 13.34	0.013

a a*R*^2^ = 0.120.

b a*R*^2^ = 0.162.

c a*R*^2^ = 0.210.

**FIG 6:**
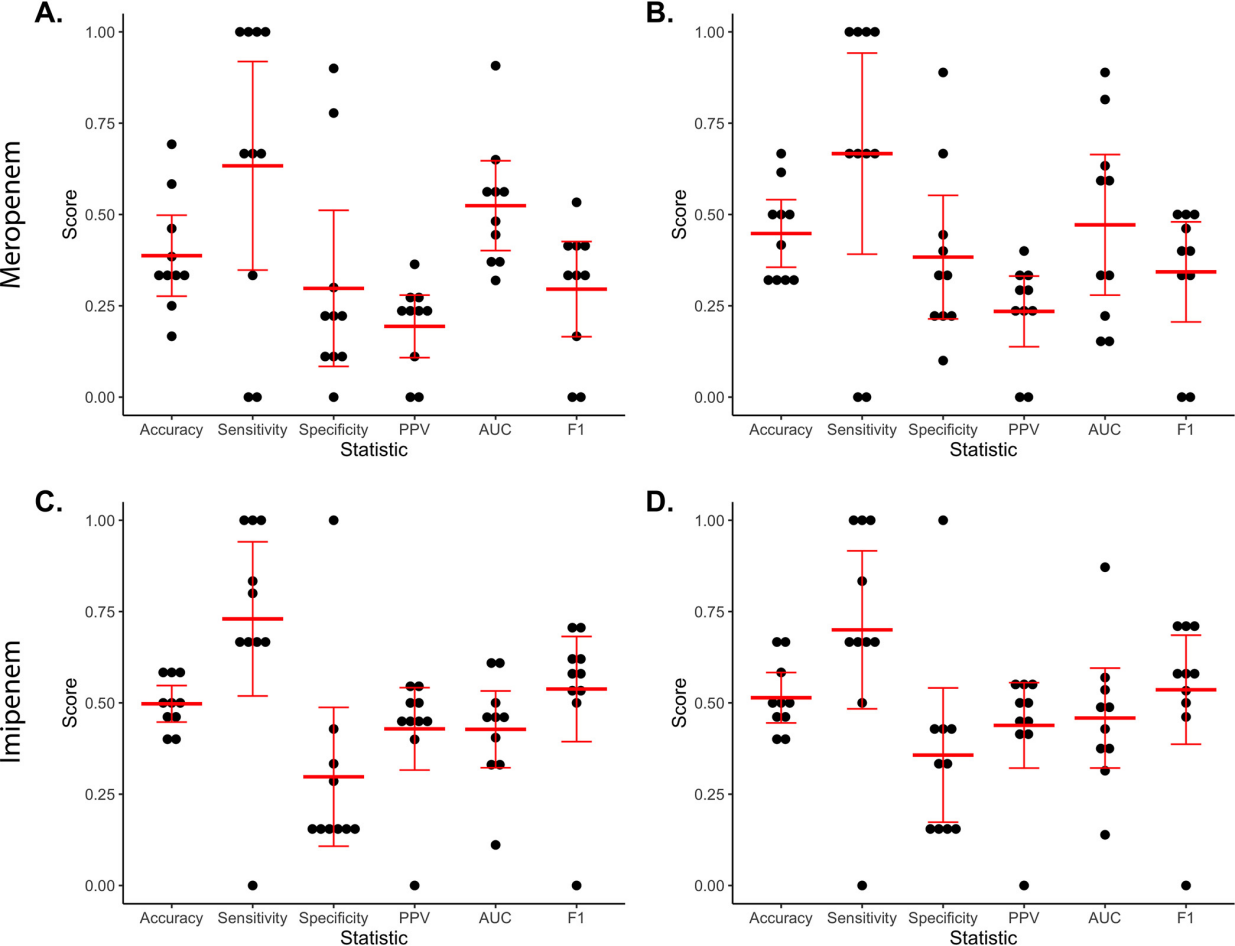
Nested cross-validation performance of the support vector classifier machine learning approach to predict high versus low carbapenem resistance among ST258 K. pneumoniae isolates (*n* = 123). Levels of meropenem and imipenem resistance were predicted using models trained on curated (A and C, respectively) and whole-genome sequence features (B and D, respectively). Accuracy, sensitivity, specificity, positive predictive value (PPV), area under the receiver operating characteristic curve (AUC), and F1 scores were determined for models built in each cross-validation fold, with mean and 95% confidence intervals displayed in red.

**(vi) Machine learning analysis.** A support vector classifier machine learning analysis was performed on the 123 ST258 isolates to predict the level of carbapenem resistance. Isolates were classified as highly resistant (MIC > 8 mg/liter) or low/intermediately resistant (MIC ≤ 8 mg/liter) to meropenem or imipenem. First, a machine learning approach was used to estimate how well high versus low resistance could be predicted based on curated features previously reported to influence carbapenem resistance: β-lactamase presence/absence, outer membrane porin phenotype, transposon isoform, and *bla*_KPC_ copy number. This showed a mean F1 score of 0.54 for imipenem and 0.30 for meropenem in nested cross-validation, which indicates relatively low predictive performances (Fig. 6A and B). We next sought to determine whether novel genetic factors not included in our curated feature set influenced resistance to carbapenems. For this purpose, we used whole-genome sequence features, including all core genome SNV loci (3,122) and all unique accessory genome elements ≥200 bp (372) within the 123 ST258 isolates. The machine learning approach here showed mean F1 scores of 0.54 for imipenem and 0.34 for meropenem in nested cross-validation, which were similar to those of the curated models (Fig. 6C and D). In summary, the support vector classifier analysis, like the regression analysis, suggested that only a small proportion of the carbapenem MIC variability in the ST258 isolates was associated with previously reported factors. Broadening the feature set to include the whole genome did not improve predictions. The use of alternative machine learning algorithms (random forest, l2-regularized logistic regression, and elastic net logistic regression) yielded similar results (data not shown).

To assess whether the poor performance of the machine learning models was the result of an insufficient sample size, we conducted learning curve analyses. For both imipenem and meropenem and for both the curated and whole-genome sequence analyses, F1 scores between the training and cross-validation sets of the support vector classifier analysis had largely converged, and cross-validation scores did not substantially increase as training set sample size increased (see Fig. S4). These findings suggest that the poor performance of the support vector classifier approach is unlikely to be caused by an insufficient sample size.

10.1128/mSystems.00194-21.5 FIG S4 Learning curves to examine how training and cross-validation F1 score varied with increasing training sample size for the support vector classifier analysis. Learning curves were constructed using curated features to predict meropenem resistance (A), whole-genome sequence features to predict meropenem resistance (B), curated features to predict imipenem resistance (C), and whole-genome sequence features to predict imipenem resistance (D). Red and green lines indicate mean F1 scores of models tested against training and cross-validation sets at each number of training examples, with the 95% confidence interval shaded. Download FIG S4, PDF file, 0.1 MB. Copyright © 2021 Bulman et al. 2021 Bulman et al. https://creativecommons.org/licenses/by/4.0/ This content is distributed under the terms of the Creative Commons Attribution 4.0 International license.

## DISCUSSION

CR-Kp causes serious infections that are associated with high mortality rates (2, 3). Increasing MICs to carbapenems are associated with worse outcomes, even among carbapenemase-producing K. pneumoniae isolates (18, 19), but the reasons for this variability are incompletely understood. Furthermore, the ability to predict carbapenem MICs in CR-Kp may also be important in some clinical settings. For example, in low- and middle-income countries where new antibiotics with activity against CR-Kp are limited or unavailable, identification of CR-Kp isolates with lower MICs to carbapenems (≤8 mg/liter) may enable the use of high-dose, prolonged infusion, carbapenem-containing combination regimens (20, 21). Various studies have shown how the presence of β-lactamases, expression levels of carbapenemase or ESBL genes, and permeability deficits, among others, can influence carbapenem activity in K. pneumoniae, but few studies have evaluated the combined effects of these genetic features on carbapenem MICs in clinical CR-Kp isolates. In the present study, multiple linear regression was used to identify significant predictors of ertapenem, imipenem, and meropenem MICs in a collection of 166 clinical CR-Kp isolates. Although the final models were only able to explain a portion of the phenotypic variability, many important and biologically plausible genotypes remained in the models, such as mutations in the outer membrane porin genes, *bla*_KPC_ copy number, and the presence of *bla*_OXA-232_.

Bioinformatic approaches are increasingly being used to predict antibiotic resistance from genomic content (22–26), and several studies have targeted K. pneumoniae and carbapenems for this purpose. Avershina and colleagues (27) applied neural network methods to β-lactamase k-mers in unassembled genomes to predict K. pneumoniae susceptibility versus resistance to carbapenems with accuracies >90%. Jaillard and colleagues used an adaptive cluster lasso algorithm to predict carbapenem susceptibility versus intermediate/resistance in K. pneumoniae with 91% to 93% accuracy (28). Nguyen and colleagues developed XGBoost-based models to predict carbapenem MICs for a collection of largely carbapenem-susceptible K. pneumoniae (29). The presence of *bla*_KPC_ and genes involved in recombination (recombinase and transposase genes) were most highly correlated with the carbapenem MICs in their model, which was accurately able to predict carbapenem MICs for >90% of isolates. Intriguingly, this approach yielded models with accuracies that remained as high as 63% to 71%, even when known antibiotic resistance genes were excluded and the interrogated gene set was limited to 100 randomly selected core genes (30). The authors speculated that their models were keying in on genomically widespread compensatory or epistatic genetic changes associated with antibiotic resistance. It is difficult to compare the results of these studies to those of our study because of their differing goals. The aims of each of these studies was to predict which K. pneumoniae isolates were susceptible and which were resistant to carbapenems. In contrast, our study focused on only carbapenem-resistant isolates and evaluated genomic features that discriminated between isolates with very high MICs and those with more modest MICs, an arguably more difficult task that may account for the poorer performance of our models.

One genotype that was associated with significant increases in the carbapenem MICs was the presence of truncated outer membrane porin channel genes. The outer membrane porins in K. pneumoniae, including OmpK35 and OmpK36, permit passage of the hydrophilic carbapenems through to their periplasmic targets. Thus, loss of function of one or multiple porin channels can diminish the activity of the carbapenems and cause low-level carbapenem resistance in the absence of carbapenemase or ESBL production (31, 32). In our cohort, *ompK35* truncation or absence was common among CR-Kp isolates (>75%), while *ompK36* truncation or absence was only noted in <5% of isolates. This is consistent with other studies that have found that *ompK35* disruption is more common among clinical isolates (33, 34). Despite its previously described association with increased carbapenem MICs, disruption of *ompK36* may be less common among clinical isolates due to fitness costs (33, 35, 36). In the present study, isolates with an absent or truncated *ompK36* gene had significantly lower imipenem MICs than isolates with OmpK36 porins that were predicted to be functional; however, these results may be an artifact of the small number of isolates with an absent or truncated *ompK36* gene in our collection. Although putative nonfunctional OmpK35 is commonly identified in CR-Kp, its role in carbapenem resistance has not been completely defined; for isolates without an ESBL or carbapenemase, some studies show that OmpK35 is important for carbapenem uptake (37), while others found no significant increase in carbapenem MIC after the loss of OmpK35 (36) if OmpK36 is still intact. The impact of having a nonfunctional OmpK35 in isolates that express an ESBL or carbapenemase is also not completely defined. Some studies have shown that nonfunctional OmpK35 porins in K. pneumoniae can contribute to carbapenem resistance for isolates that express KPC (33) or ESBL enzymes (34). Interestingly, we found that ertapenem, imipenem, and meropenem MICs were each significantly increased in isolates with truncated *ompK35* genes, most of which contained a carbapenemase gene and an intact *ompK36* gene. This further suggests that *ompK35* mutations may in fact confer increased carbapenem resistance in isolates that express a carbapenemase.

Another outer membrane porin, OmpK37, is often expressed in isolates that are deficient in OmpK35 and OmpK36 but is usually downregulated when these porins are expressed (38). OmpK37 enables the passage of carbapenems, though it may not as readily permit other β-lactams to cross the membrane (31, 38, 39). Herein, we found that CR-Kp isolates with a mutation in *ompK37* of unclear functional significance had lower carbapenem MICs than isolates with a wild-type copy of *ompK37.* For example, the wild-type *ompK37* allele was found to increase the meropenem MIC in its final linear regression model. There are at least two potential explanations for this association between *ompK37* mutations and lower carbapenem MICs. First, mutations that impact the structure of the porin may enhance permeability to the β-lactams and could be responsible for the observed differences in carbapenem MICs (31, 40). Previous studies found that nonsynonymous mutations in the constriction region of the OmpK35 homolog OmpF increased or decreased the permeability to various cephalosporins and ampicillin (41, 42). Others have suggested that variations in the external loops of the porin channels in K. pneumoniae may impact permeability (31). Mutations in other regions of the porins, including the β-strands, have been shown to alter the electrostatic field of the pore and modify β-lactam passage in clinical isolates (43). In the present study, the two mutations observed in the CR-Kp isolates with lower carbapenem MICs were in β-strands that cross the outer membrane. Further investigation is required to determine if the mutations we observed in *ompK37* can enhance carbapenem penetration. A second potential explanation, which is not necessarily mutually exclusive, is that isolates with these mutations are more likely to have wild-type *ompK35* and/or *ompK36* genes due to selective pressures that require CR-Kp to maintain some functional porin channels for adequate transport of nutrients. In the present study, isolates with mutations in *ompK37* were approximately 80% more likely to coharbor functional *ompK35* genes but less likely to harbor functional *ompK36* genes than isolates with wild-type *ompK37*. Thus, isolates with mutations in *ompK37* may on average also have lower carbapenem MICs due to increased drug passage through alternative porin channels. Future studies should also include functional characterization and assess expression of the porin channels to confirm or refine these proposed hypotheses.

In the present study, most CR-Kp isolates had a carbapenemase gene, and *bla*_KPC_ was the most common. The *bla*_KPC_ gene copy number was significantly correlated with the carbapenem MICs and was retained in all three multivariate models. However, this explained only a small portion of the variation in carbapenem MICs. Previous studies have likewise found that *bla*_KPC_ gene expression does not always highly correlate with the gene copy number and is also influenced by differences in the upstream region of the gene and transcriptional start site (7, 44). Outer membrane porin mutations in combination with higher KPC expression may further increase the carbapenem MICs (7). The combination of outer membrane porin mutations and elevated *bla*_KPC_ gene copy number has also been observed in KPC-Kp isolates that are resistant to meropenem-vaborbactam (45) and ceftazidime-avibactam (8). In contrast to this correlation with *bla*_KPC_ gene copy number, the presence of a *bla*_ESBL_ gene did not significantly increase carbapenem MICs in our collection of CR-Kp isolates. Previous studies have shown that *bla*_ESBL_ genes such as *bla*_CTX-M_ or *bla*_SHV-5_ in combination with outer membrane porin deficits can cause carbapenem resistance in K. pneumoniae isolates that do not coharbor carbapenemase genes (13, 14). Similarly, *bla*_ESBL_ gene duplications have been shown to increase carbapenem MICs in isolates without a carbapenemase (46). However, the present study suggests, for the first time, that neither the presence of an *bla*_ESBL_ nor the *bla*_ESBL_ gene copy number appreciably increases the carbapenem MICs in isolates that coharbor carbapenemase genes, which is supported by the absence of these genomic features in the final multivariate models.

The *bla*_KPC_ gene is typically found in the Tn*4401* transposon of *Enterobacterales* isolates from the United States and Europe, while other transposons such as the Tn*3*-Tn*4401* chimera are more widespread in China (11, 47). Isoforms of Tn*4401* have also been characterized with variations in the promoters that are present upstream of the *bla*_KPC_ gene, leading to differences in *bla*_KPC_ gene expression and β-lactam resistance. For example, the presence of the *bla*_KPC_ gene in the Tn*4401a* variant (P1 and P2 promoters) has been shown to lead to higher *bla*_KPC_ expression than Tn*4401b* (P1, P2, and P3 promoters), thereby increasing carbapenem MICs 4- to 8-fold (11, 12). The most common Tn*4401* transposon variants in the *bla*_KPC_-harboring CR-Kp isolates in the present study were Tn*4401b* and Tn*4401d* (P1 and P2 promoters). Naas et al. previously showed that stronger *bla*_KPC_ expression occurs when only the P1 and P2 promoters are present, and this can subsequently increase carbapenem MICs (12). Thus, since Tn*4401d* has only the P1 and P2 promoters, while Tn*4401b* has all three promoters, isolates with Tn*4401d* may have higher *bla*_KPC_ expression than those with Tn*4401b*. The phenotypic data from our clinical *bla*_KPC_-harboring isolates corresponded relatively well with the findings from this previous study, as we observed that isolates with Tn*4401d* had higher mean carbapenem MICs than isolates with Tn*4401b.* However, transposon isoforms were not found to be significantly associated with the carbapenem MICs in the multivariate models.

Regression analysis indicated that factors known to impact carbapenem MICs accounted for a relatively small portion of the variability we observed in our cohort. To determine whether as-yet-unidentified genetic features were also associated with increased or decreased carbapenem MICs, we employed an agnostic machine learning approach that interrogated the entire genomes of the ST258 isolates. Surprisingly, incorporating core genome SNVs and accessory genomic elements failed to better predict highly resistant strains than when solely using known factors. Several explanations may account for this. Variables not included in our whole-genome feature set, such as *bla*_KPC_ copy number, epigenetic changes, or subtle differences between homologous accessory genes, may play a large role in dictating carbapenem MICs. The majority of highly resistant isolates may each have unique MIC-altering SNV changes not shared with other isolates, making the machine learning algorithm “blind” to them. Alternatively, variability in MIC measurements (e.g., intrinsic Etest variation, instability of MICs over time, or irregularities caused by heteroresistance) may have obfuscated associations between degree of carbapenem resistance and genetic features. In future studies, these possibilities will need to be examined to gain a more thorough understanding of how CR-Kp resists killing by carbapenems.

One important limitation that may have impacted univariate and multivariate analyses of our study was that carbapenem MICs were only evaluated to a maximum concentration of 32 mg/liter. Thus, any isolate with an MIC >32 mg/liter was assigned an MIC of 64 mg/liter for statistical evaluations even though these CR-Kp isolates may in actuality have differing levels of carbapenem resistance. More precisely defined carbapenem MICs may affect the results of these analyses. Another potential limitation is that the isolates were collected from a single institution, which may impact the ability to extrapolate our findings to other medical centers with differing CR-Kp genotypes. However, the general characteristics of our CR-Kp collection appear mostly representative of resistant isolates found by other investigators, at least in the United States (2, 48). An additional limitation is that short-read sequencing was used to genomically characterize all isolates. For this reason, we were not able to define plasmid sequences, and novel plasmid insertions, deletions, and rearrangements capable of affecting *bla*_KPC_ expression may have been missed. In future studies, long-read sequencing will be performed on these isolates to better characterize the plasmids they carry. Lastly, the carbapenem MICs were collected via Etest, which is generally reliable for determining categorical resistance in carbapenemase-producing K. pneumoniae but may not define the actual MIC as accurately as broth microdilution (49). Thus, our findings may not necessarily be generalizable to MIC data derived from alternative testing methods. However, since Etest MICs were used for all analyses, relative interisolate MIC differences are likely accurate at least for this testing method.

In conclusion, we collected and conducted an in-depth molecular analysis of 166 CR-Kp isolates. Multiple linear regression models identified genotypes that were associated with significant changes in carbapenem MICs. The models support the roles of several biologically plausible genotypes in contributing to carbapenem MICs among clinical CR-Kp isolates, including *bla*_KPC_ copy number and outer membrane porins. In contrast, the presence of ESBL genes or their increased copy number were not associated with carbapenem MICs, suggesting they do not increase carbapenem resistance among isolates that already harbor a carbapenemase gene. Lastly, our data also showed a trend toward increased carbapenem MICs among isolates with Tn*4401d.* Machine learning analyses did not substantially improve the ability to predict isolates with high or low MICs based on their sequence. Future studies are necessary to refine the models and fully define the role of these genes in carbapenem resistance.

## MATERIALS AND METHODS

### Clinical isolate collection and carbapenem resistance testing.

A retrospective analysis was performed on 174 CR-Kp isolates that were recovered as a part of routine clinical care. The isolates were collected from consecutive patients at a tertiary care hospital in the United States (Northwestern Memorial Hospital, Chicago, IL) between October 2010 and February 2016. Carbapenem resistance was defined as displaying resistance to ≥1 carbapenem following MIC testing with a Vitek 2 system and using CLSI breakpoints (50, 51). CR-Kp isolates were archived and stored at −80°C by the hospital microbiology laboratory as part of the routine institutional infection control policy. Isolates were subsequently recovered by streaking onto Luria-Bertani (LB) solid medium and incubating at 37°C overnight. To ensure purity, a single colony was selected from the plate for cryopreservation. Isolates were regrown on LB agar, and MICs to meropenem, ertapenem, and imipenem were measured using Etest strips (bioMérieux) according to the manufacturer’s specifications to confirm carbapenem resistance. Isolates that were not resistant to at least 1 carbapenem following the Etest were excluded from the study. Observed MIC values were rounded up to the next highest log_2_ dilution per the manufacturer’s specifications. Off-scale values (i.e., MICs >32 mg/liter) were converted to 64 mg/liter for statistical analyses. Only the first isolate per patient was included. Isolates recovered from rectal swabs were also excluded from this study. This study was approved by the Northwestern University Institutional Review Board (IRB). A waiver of informed consent was obtained due to the retrospective nature of the study. No diagnostic or treatment decisions were affected by this study.

### Whole-genome sequencing.

Isolates were grown overnight in LB broth with shaking at 37°C. DNA extraction was performed using a Maxwell 16 instrument with cell DNA purification kits (Promega Corporation, Madison, WI) according to the manufacturer’s instructions. Libraries were prepared using the Illumina Nextera XT kit and sequenced using an Illumina HiSeq (150-bp paired-end reads) platform (Illumina, Inc., San Diego, CA). Sequences were trimmed using Trimmomatic v0.32 (51), and then *de novo* assembly was performed with SPAdes 3.9.1. Contigs were removed if they were shorter than 200 bp or had a mean fold coverage of <5× per base.

### CR-Kp genome analysis.

*In silico* multilocus sequence typing (MLST) was performed by analyzing each assembled sequence with MLST 1.8 (Center for Genomic Epidemiology) (52). The capsule locus type was determined by analyzing the genome of each isolate in Kaptive, and calls with confidence levels of “good” or better were reported (53). The antimicrobial resistance genes were identified by aligning the assembled CR-Kp genomes against the Comprehensive Antibiotic Resistance Database (54) and ResFinder v3.1 (55). Whole-genome sequencing reads were aligned to the ST258 reference genome KPNIH1 (NZ_CP008827.1) using bwa v0.7.15. This sequence was chosen as the reference as it is a publicly available closed ST258 genome. Variants relative to the reference sequence were determined using bcftools v1.9 using a minimum base quality of 25, minimum mapping quality of 30, and a haploid model as previously described (56). Only variants with a minimum SNV quality score of 200, minimum read consensus of 75%, and a minimum read depth of 5 with at least 1 read in each direction were kept. A genome alignment was created from variant positions relative to the KPNIH1 reference genome using bcftools_filter_and_align (https://github.com/egonozer/bcftools_filter/blob/master/bcftools_filter_and_align.pl). FastTree v2.1.9 was used to construct the maximum likelihood phylogenetic tree from the resulting alignment using the generalized time-reversible model with branch rescaling to optimize the Gamma20 likelihood. Phylogenetic trees were visualized using FigTree v1.4.4 and R v4.0.2 with ggtree package v2.2.4. To examine whether the *bla*_SHV_ (ESBL), *bla*_CTX-M_, and *bla*_KPC_ gene copy numbers contributed to elevated carbapenem MICs, the average number of β-lactamase sequencing reads were normalized to the total number of core genome sequencing reads for each isolate using sam_to_coverage (https://github.com/egonozer/sam_to_coverage/blob/master/sam_to_coverage.pl); the ratio was used to predict the copy numbers of the β-lactamase genes relative to the core genome copy number. To distinguish between duplication of the *bla*_KPC_ gene itself or increased copy number of the entire plasmid carrying *bla*_KPC_, we compared the average read coverage of *bla*_KPC_ to the average read coverages of the other 5 genes on the *bla*_KPC-3_-carrying pRYCKPC3.1 plasmid (NC_019151.1). The isoforms of the transposons harboring the *bla*_KPC_ genes were analyzed by aligning the sequences upstream of the *bla*_KPC_ genes to published reference sequences (12). Mutations in the transposons were annotated based on the Tn*4401b* reference sequence from pKPC_UVA01 (accession CP017937), as previously described (57). The sequences of the outer membrane porin channel genes from each isolate were examined to predict if they encoded functional porin channels. Isolates with *ompK35*, *ompK36*, and *ompK37* alleles that were absent or had a premature stop codon or insertion sequence in the gene were considered to produce an absent or truncated porin channel, whereas isolates with genes that were identical to the *ompK35* (accession AJ011501), *ompK36* (accession JX310551, Z33506, AP006725, and JOOW00000000), and *ompK37* (accession KC534871 and NZ_LR130541) reference sequences from isolates with a known functional OmpK protein were predicted to have functional porin channels. Isolates with nonsynonymous mutations in porin channel genes that did not lead to a premature stop codon were separately categorized as having unknown function.

### Data and regression analyses.

To determine if variations in genotype (predicted OmpK function, ESBL presence, and transposon isoform) were associated with differences in phenotype (carbapenem MIC), univariate analyses were performed and data were compared via the Mann-Whitney U test. Nonparametric continuous data are presented as the median, and a *P* value of ≤0.05 was considered statistically significant. To determine the strength and direction of the relationship between the relative *bla*_KPC_ copy number and the carbapenem MICs, Spearman’s correlation analysis was performed. The continuous variable *bla*_KPC_ copy number was dichotomized at <4 and ≥4 to compare the isolates with the highest MIC *bla*_KPC_ copy number (>1 standard deviation above mean) to the rest of the CR-Kp isolates based on the observed distribution among the sample (mean ± standard deviation [SD]: 2.70 ± 1.38).

To determine the combined influence of various clinically relevant genes on the ertapenem, imipenem, and meropenem MICs, multiple linear regression via a stepwise approach was utilized to evaluate the incremental change in the phenotypic carbapenem MIC associated with one or more identified genotypic markers of interest. Carbapenem MICs were modeled as observed from the Etest (i.e., 2, 4, 8, and 16 mg/liter). Any genomic element identified in >1 isolate was eligible for model inclusion based on prevalence, biologic plausibility, and/or previous studies. Thus, the candidate variables (genotype) initially considered were the following: *bla*_AmpC_ presence, *bla*_CTX-M_ presence, *bla*_CTX-M_ copy number, *bla*_KPC_ presence, *bla*_KPC_ copy number, *bla*_FOX-5_ presence, *bla*_OXA-232_ presence, *bla*_OXA_ (noncarbapenemase) presence, *bla*_SHV_ (non-ESBL) presence, *bla*_SHV_ (ESBL) presence, *bla*_SHV_ (ESBL) copy number, *bla*_TEM_ presence, *bla*_VEB-9_ presence, predicted function of *ompK35/36/37*, and transposon isoform. A *P* value of ≤0.05 was considered statistically significant in the final model. Model performance was evaluated using adjusted *R*^2^ (a*R*^2^). Collinearity was assessed via tolerance and variance inflation factor. Outliers, highly influential values, and leverage points were not removed since they were true observed values. Multiple linear regression analyses were performed using SPSS version 26 (IBM Corporation, Armonk, NY).

### Machine learning analysis.

Machine learning analyses were performed on the ST258 CR-Kp isolates (*n* = 123) using the sci-kit learn library (v0.23.2) (https://www.jmlr.org/papers/v12/pedregosa11a.html) in Python v3.6.12 following the pipeline described by Pincus et al. (58) For the curated analysis, β-lactamase presence/absence, outer membrane porin phenotype, transposon isoform, and *bla*_KPC_ copy number were used as features and converted to binary variables. For example, *bla*_KPC_ copy number was considered “high” if ≥ 4 copies were present and “low” if < 4 copies were present. Features present in all or none of the isolates (e.g., *bla*_NDM_) were removed. For the whole-genome sequence analysis, all core genome (defined as sequences present in ≥95% of the 123 ST258 isolates) SNVs and accessory genomic elements (AGEs) of ≥200 bp were used as features. To define core genome SNVs, alignments of ST258 genomes to the KPNIH1 reference genome were created as described above, noncore loci were masked, and invariant sites were removed. AGEs in the ST258 genomes were defined using Spine v0.32, AGEnt v0.31, and ClustAGE v0.8 (59, 60), grouping together perfectly correlated accessory sequences. As part of the analysis pipeline, SNV loci were converted to binary features through one-hot-encoding.

The ability of machine learning models built using curated or whole-genome sequence features to predict whether isolates had high resistance (MIC > 8 mg/liter) or low resistance (MIC ≤ 8 mg/liter) to imipenem or meropenem was estimated using a nested cross-validation approach. The support vector classifier algorithm was used with C, gamma, and class_weight included as hyperparameters. F1 scores (0, lowest predictive power; 1, highest predictive power) were used as the primary scoring metric. In the outer 10-fold stratified cross-validation loop, training data for each fold were used to build a model that was evaluated against the held-out test set. Grid-search cross-validation (the inner loop) was used to select hyperparameters for each model. In addition to the F1 score, accuracy, sensitivity, specificity, positive predictive value, and area under the receiver operating characteristic curve were also calculated for each model. Learning curves were created to examine the impact of increasing training sample size on training and cross-validation F1 score. For each combination of features (curated and whole-genome sequence) and labels (meropenem and imipenem resistance), the data set was split into training and cross-validation folds through 10-fold stratified cross-validation. Subsets of examples from each training fold were used to train models with hyperparameters selected via grid search cross-validation. Training and cross-validation F1 scores were then calculated at each training sample size. Alternative machine learning algorithms (random forest, l2-regularized logistic regression, and elastic net logistic regression) were used following a similar approach. Code for the machine learning analyses are available on GitHub (https://github.com/nathanpincus/CRKP_Resistance_Prediction).

### Data availability.

Sequencing reads and assembled genomes have been submitted to GenBank under BioProject PRJNA395086.
