# Supplementary material for: Genomic Features Associated with the Degree of Phenotypic Resistance to Carbapenems in Carbapenem-Resistant Klebsiella pneumoniae
Source: mSystems. 2021 Sep 14;6(5):e00194-21. doi: 10.1128/mSystems.00194-21 (PMC8547452; doi:10.1128/mSystems.00194-21)
Supplement: TABLE S1 [file msystems.00194-21-st001.pdf]

Isolate ID	Number of Bases	Number of Reads	Number of Assembled Contigs	Assembly Size (bp)	N50	%GC	Fold Coverage	MLST	Capsule Locus Type	Clinical Source	Meropenem Etest MIC (mg/L)	Ertapenem Etest MIC (mg/L)	Imipenem Etest MIC (mg/L)
**CRE-002**	1,087,056,248	7,199,048	438	5,830,037	139,053	57.06	186	ST258	KL107	Respiratory Tract	>32	>32	>32
**CRE-004**	884,570,382	5,858,082	885	6,088,375	130,289	56.89	145	ST258	KL107	Respiratory Tract	8	16	8
**CRE-007**	1,077,091,456	7,133,056	406	5,943,127	129,657	56.92	181	ST258	KL107	Urine	4	4	4
**CRE-008**	1,171,628,932	7,759,132	826	6,130,313	142,585	56.68	191	ST258	KL107	Urine	2	2	16
**CRE-010**	672,466,892	2,846,990	131	5,865,820	261,970	57.01	115	ST258	KL107	Respiratory Tract	16	>32	2
**CRE-013**	393,758,774	2,607,674	768	6,136,045	134,034	56.78	64	ST258	KL107	Urine	4	8	8
**CRE-017**	262,973,525	925,456	152	5,466,294	118,117	57.39	48	ST45		Urine	1	4	0.5
**CRE-019**	506,449,772	3,353,972	685	6,033,415	130,289	56.86	84	ST258	KL107	Respiratory Tract	16	4	>32
**CRE-023**	780,199,484	5,166,884	478	5,947,503	130,289	56.96	131	ST258		Respiratory Tract	16	>32	>32
**CRE-025**	342,298,276	2,266,876	522	5,982,364	134,024	56.89	57	ST258	KL107	Urine	8	8	>32
**CRE-027**	597,868,494	3,959,394	530	5,928,149	129,689	56.95	101	ST258	KL107	Respiratory Tract	>32	16	>32
**CRE-028**	320,664,204	2,123,604	419	5,890,367	124,373	57	54	ST258		Urine	>32	8	16
**CRE-032**	569,138,026	3,769,126	407	5,854,705	130,663	56.99	97	ST258	KL107	Urine	8	>32	8
**CRE-035**	911,223,996	6,034,596	611	6,173,324	129,657	56.76	148	ST258	KL107	Respiratory Tract	4	8	8
**CRE-036**	470,872,964	3,118,364	323	5,645,021	204,945	57.06	83	ST22	KL9	Other	4	>32	1
**CRE-038**	2,211,359,968	14,644,768	1737	6,325,300	126,617	56.68	350	ST258	KL107	Respiratory Tract	4	16	8
**CRE-041**	945,915,944	6,264,344	443	5,629,026	149,643	57.18	168	ST258	KL106	Respiratory Tract	16	>32	8
**CRE-043**	900,752,146	5,965,246	381	5,956,021	137,721	56.95	151	ST258	KL107	Other	>32	>32	8
**CRE-044**	1,048,020,030	6,940,530	432	5,960,571	130,583	56.93	176	ST258	KL107	Urine	8	16	16
**CRE-046**	318,120,156	2,106,756	288	5,905,577	126,085	56.96	54	ST258		Respiratory Tract	16	>32	8
**CRE-047**	555,236,060	3,677,060	450	5,999,872	126,389	56.89	93	ST258	KL107	Urine	>32	>32	>32
**CRE-048**	743,305,654	4,922,554	467	5,960,406	130,289	56.91	125	ST258		Respiratory Tract	8	>32	8
**CRE-049**	683,017,092	4,523,292	356	6,096,338	119,157	56.8	112	ST258	KL107	Blood	4	8	16
**CRE-050**	505,117,046	3,345,146	731	5,986,202	143,063	56.7	84	ST258	KL107	Urine	>32	>32	4
**CRE-051**	399,639,318	2,646,618	725	6,040,381	137,377	56.9	66	ST258	KL107	Respiratory Tract	8	8	16
**CRE-052**	594,471,296	3,936,896	784	6,044,682	127,177	56.96	98	ST258		Urine	4	4	16
**CRE-055**	553,341,312	3,664,512	223	5,640,769	114,430	56.96	98	ST13	KL3	Urine	16	>32	>32
**CRE-056**	668,358,918	4,426,218	408	6,117,687	137,517	56.79	109	ST258	KL107	Respiratory Tract	4	16	8
**CRE-057**	665,928,724	4,410,124	345	6,091,621	130,289	56.81	109	ST258		Respiratory Tract	16	>32	8
**CRE-058**	549,903,646	3,641,746	568	5,593,968	330,202	57.3	98	ST16		Respiratory Tract	1	2	0.25
**CRE-059**	474,118,860	3,139,860	619	6,131,166	110,178	56.82	77	ST258	KL107	Urine	4	8	16
**CRE-060**	562,079,078	3,722,378	590	6,005,941	127,177	56.64	94	ST258	KL107	Urine	4	8	8
**CRE-061**	400,745,143	1,692,836	183	5,920,776	216,118	56.95	68	ST258	KL107	Respiratory Tract	4	16	16
**CRE-062**	597,256,340	3,955,340	350	5,972,660	126,390	56.91	100	ST258	KL107	Urine	>32	>32	>32
**CRE-065**	520,582,444	2,284,550	150	5,798,132	210,520	56.47	90	ST15	KL24	Respiratory Tract	2	4	4
**CRE-066**	404,006,238	2,675,538	529	5,558,650	285,386	57.3	73	ST16		Urine	0.5	2	0.25
**CRE-067**	432,291,256	2,862,856	524	5,948,357	123,851	57.01	73	ST258	KL107	Urine	8	8	8
**CRE-068**	463,543,238	1,933,066	134	5,837,225	334,970	56.77	79	ST101	KL17	Respiratory Tract	4	>32	1
**CRE-070**	516,296,642	2,174,994	182	5,960,167	214,747	56.92	87	ST258	KL107	Urine	>32	8	>32
**CRE-074**	367,106,670	2,431,170	473	5,900,189	133,091	56.97	62	ST258	KL107	Other	16	>32	>32
**CRE-078**	384,320,972	2,545,172	357	5,938,364	134,096	56.94	65	ST258	KL107	Respiratory Tract	8	8	>32
**CRE-081**	399,576,804	2,646,204	320	5,840,836	129,666	57.01	68	ST258		Respiratory Tract	2	16	4
**CRE-082**	650,538,200	4,308,200	389	5,874,685	130,660	56.99	111	ST258	KL107	Urine	4	>32	8
**CRE-083**	371,784,348	2,462,148	254	5,531,745	130,048	57.18	67	ST15	KL112	Respiratory Tract	4	>32	4
**CRE-085**	2,472,963,240	16,377,240	747	5,799,170	141,982	57.14	426	ST258	KL107	Urine	4	16	16
**CRE-087**	906,804,226	6,005,326	289	5,864,216	129,657	56.98	155	ST258	KL107	Blood	4	16	4
**CRE-088**	661,310,842	4,379,542	207	5,872,449	134,034	56.98	113	ST258	KL107	Urine	2	8	16
**CRE-089**	1,227,355,180	8,128,180	312	5,908,592	127,177	56.98	208	ST258	KL107	Urine	2	>32	>32
**CRE-091**	790,966,388	5,238,188	539	5,744,104	124,389	56.88	138	ST15		Respiratory Tract	2	8	16
**CRE-094**	552,449,705	2,214,564	140	5,711,058	207,238	57.16	97	ST258	KL107	Respiratory Tract	2	4	>32
**CRE-095**	414,199,256	1,619,766	168	5,942,516	214,747	56.91	70	ST258	KL107	Urine	4	>32	>32
**CRE-096**	1,907,524,110	12,632,610	460	5,921,464	128,890	56.96	322	ST258	KL107	Respiratory Tract	8	>32	>32
**CRE-098**	505,393,376	3,346,976	232	5,641,727	114,430	56.96	90	ST13	KL3	Respiratory Tract	8	8	8
**CRE-099**	392,179,012	2,597,212	258	6,130,547	121,578	56.67	64	ST258	KL107	Urine	4	4	8

**CRE-100**	450,990,446	1,893,792	121	5,941,406	231,253	56.8	76	ST258	KL107	Urine	4	8	8
**CRE-101**	348,551,146	1,559,080	196	5,624,832	179,578	57.22	62	ST1082		Other	>32	>32	4
**CRE-102**	914,755,584	6,057,984	203	5,916,702	130,290	56.57	155	ST258	KL107	Urine	4	8	>32
**CRE-103**	408,284,370	2,703,870	261	5,733,450	137,468	57.13	71	ST258	KL107	Urine	>32	>32	>32
**CRE-104**	939,485,760	6,221,760	634	5,843,800	133,969	57.08	161	ST258	KL107	Urine	8	>32	8
**CRE-105**	1,547,836,976	10,250,576	1000	6,127,472	127,051	56.9	253	ST258	KL107	Other	>32	>32	>32
**CRE-106**	1,581,021,038	10,470,338	588	5,965,199	152,566	56.67	265	ST258	KL107	Urine	>32	>32	16
**CRE-107**	1,316,110,564	8,715,964	518	5,930,859	143,715	56.76	222	ST258	KL107	Other	8	>32	4
**CRE-109**	1,086,579,390	7,195,890	2505	6,499,957	153,939	56.79	167	ST45	KL24	Urine	4	>32	4
**CRE-110**	987,125,052	6,537,252	935	6,049,125	129,689	56.78	163	ST258	KL107	Urine	1	4	2
**CRE-112**	1,345,547,108	8,910,908	773	5,983,015	130,289	56.93	225	ST258	KL107	Urine	8	>32	8
**CRE-113**	1,095,183,672	7,252,872	493	6,032,044	130,603	56.87	182	ST258	KL107	Respiratory Tract	16	>32	16
**CRE-114**	1,387,126,468	9,186,268	763	6,275,227	137,534	56.27	221	ST258	KL107	Urine	1	2	1
**CRE-115**	1,782,092,638	11,801,938	559	6,179,500	129,689	56.45	288	ST258		Urine	8	16	8
**CRE-117**	1,044,064,736	6,914,336	413	5,945,019	152,205	56.95	176	ST258	KL107	Urine	8	>32	16
**CRE-121**	863,703,692	5,719,892	577	5,922,606	137,623	57.03	146	ST258		Urine	2	>32	1
**CRE-122**	975,668,380	6,461,380	673	5,792,324	133,091	57.13	168	ST258	KL107	Urine	4	8	4
**CRE-123**	1,105,997,688	7,324,488	447	5,868,832	141,981	57.03	188	ST258	KL107	Urine	2	8	2
**CRE-124**	978,635,530	6,481,030	782	5,833,554	132,785	57.06	168	ST258		Respiratory Tract	4	8	4
**CRE-127**	1,336,171,518	8,848,818	679	5,862,519	170,342	56.98	228	ST258	KL107	Respiratory Tract	8	8	16
**CRE-129**	1,315,410,226	8,711,326	169	5,609,304	130,499	57.22	235	ST258	KL107	Blood	2	4	4
**CRE-130**	1,102,598,376	7,301,976	424	5,876,885	137,330	56.98	188	ST258		Urine	4	4	1
**CRE-131**	1,263,933,118	8,370,418	363	5,859,009	154,281	57.03	216	ST258		Urine	8	>32	8
**CRE-132**	1,097,864,828	7,270,628	256	5,784,018	137,517	57.12	190	ST258		Blood	4	>32	4
**CRE-133**	547,355,370	3,624,870	258	5,690,723	139,053	57.16	96	ST258	KL107	Urine	2	8	2
**CRE-135**	367,017,580	2,430,580	584	5,701,851	88,995	57.08	64	ST15	KL112	Urine	2	4	1
**CRE-136**	615,311,108	4,074,908	155	6,010,639	130,292	56.73	102	ST258	KL107	Respiratory Tract	4	8	2
**CRE-137**	555,407,899	3,725,920	245	5,875,850	220,206	57.07	95	ST258	KL107	Urine	4	8	16
**CRE-138**	609,201,011	4,122,634	230	5,714,171	204,099	57.14	107	ST15		Respiratory Tract	2	2	4
**CRE-141**	1,366,338,298	9,048,598	326	6,084,452	126,389	56.81	225	ST258	KL107	Blood	4	>32	2
**CRE-143**	1,529,438,834	10,128,734	315	6,009,706	152,205	56.74	254	ST258	KL107	Urine	1	4	2
**CRE-152**	837,281,410	5,544,910	214	5,818,151	134,034	57.05	144	ST258	KL107	Other	8	>32	2
**CRE-153**	773,125,436	5,120,036	339	5,887,904	130,143	56.99	131	ST258	KL107	Urine	4	>32	4
**CRE-154**	841,959,692	5,575,892	385	5,981,901	150,209	56.79	141	ST258	KL107	Urine	1	8	1
**CRE-155**	752,667,654	4,984,554	293	5,835,383	151,720	56.94	129	ST258		Other	8	>32	4
**CRE-156**	851,561,782	5,639,482	460	5,657,046	181,486	57.14	151	ST45	KL24	Respiratory Tract	1	8	0.5
**CRE-157**	797,408,350	5,280,850	263	5,796,313	134,034	57.08	138	ST258	KL107	Urine	2	4	1
**CRE-160**	547,460,164	3,625,564	464	5,962,832	130,143	56.97	92	ST258	KL107	Urine	8	8	16
**CRE-161**	834,055,748	5,523,548	344	5,940,379	137,925	56.99	140	ST258	KL107	Blood	4	8	8
**CRE-163**	792,029,428	5,245,228	399	5,442,788	128,701	57.3	146	ST15		Urine	2	4	4
**CRE-165**	886,024,210	5,867,710	189	5,850,305	134,034	57.01	151	ST258	KL107	Respiratory Tract	>32	>32	>32
**CRE-166**	1,079,489,034	7,148,934	565	5,992,407	129,666	56.95	180	ST258	KL107	Respiratory Tract	4	>32	>32
**CRE-167**	1,765,511,932	11,692,132	445	5,930,523	130,288	56.85	298	ST258	KL107	Urine	4	8	16
**CRE-168**	728,637,816	4,825,416	135	5,889,840	137,534	56.84	124	ST258	KL107	Urine	4	8	8
**CRE-170**	490,031,844	3,245,244	282	5,560,330	135,548	57.16	88	ST15	KL24	Respiratory Tract	8	16	2
**CRE-171**	604,465,080	4,003,080	223	5,902,594	126,755	56.83	102	ST258		Respiratory Tract	4	>32	8
**CRE-172**	641,533,768	4,248,568	337	5,960,982	129,666	56.91	108	ST258	KL107	Urine	4	8	8
**CRE-173**	447,046,070	2,960,570	247	5,846,089	108,921	57	76	ST258	KL107	Other	16	16	4
**CRE-176**	464,835,371	2,298,542	130	5,728,851	178,833	57.11	81	ST258	KL107	Blood	8	16	16
**CRE-177**	378,823,666	2,508,766	367	5,681,654	185,613	57.17	67	ST16	KL149	Blood	1	16	1
**CRE-178**	684,408,406	4,532,506	351	6,153,620	143,116	56.4	111	ST258	KL107	Urine	2	8	2
**CRE-180**	751,083,362	4,974,062	367	5,815,749	127,177	57.05	129	ST258	KL107	Urine	1	1	8
**CRE-183**	846,111,588	5,603,388	233	5,917,303	149,643	56.82	143	ST258	KL107	Other	2	8	4
**CRE-186**	658,152,828	4,358,628	350	5,685,760	125,025	57.03	116	ST15	KL112	Urine	1	2	16
**CRE-188**	673,194,844	4,458,244	440	5,999,130	159,269	56.79	112	ST258	KL107	Urine	2	>32	8
**CRE-190**	694,195,018	4,597,318	463	5,672,156	134,017	57	122	ST15		Respiratory Tract	2	4	2
**CRE-191**	935,105,250	6,192,750	332	5,903,379	149,643	56.82	158	ST258	KL107	Urine	4	>32	>32

**CRE-194**	658,783,404	4,362,804	197	5,550,133	146,915	57.22	119	ST15	KL24	Respiratory Tract	>32	>32	>32
**CRE-195**	553,085,820	3,662,820	230	5,893,941	222,110	56.91	94	ST14	KL2	Urine	1	4	8
**CRE-196**	735,128,400	4,868,400	227	5,955,403	137,516	56.98	123	ST258	KL107	Urine	8	>32	16
**CRE-197**	489,831,316	3,243,916	411	5,511,231	54,664	57.43	89	ST16		Urine	1	2	16
**CRE-201**	928,797,980	6,150,980	351	5,899,700	126,914	57.01	157	ST258	KL107	Urine	4	>32	8
**CRE-203**	1,079,010,666	7,145,766	290	5,747,736	311,126	56.8	188	ST955	KL3	Urine	4	8	4
**CRE-204**	1,027,076,632	6,801,832	289	5,546,725	152,206	57.19	185	ST340	KL15	Other	0.5	2	1
**CRE-205**	916,768,716	6,071,316	410	5,802,471	126,390	56.98	158	ST258	KL107	Respiratory Tract	4	>32	16
**CRE-206**	1,170,880,274	7,754,174	360	5,953,894	149,643	56.8	197	ST258	KL107	Urine	4	>32	>32
**CRE-207**	1,344,362,362	8,903,062	404	5,962,635	143,817	56.78	225	ST258	KL107	Urine	8	>32	>32
**CRE-208**	1,036,328,402	6,863,102	201	5,742,084	227,757	57.14	180	ST14	KL2	Urine	4	8	8
**CRE-209**	800,983,728	5,304,528	189	5,705,308	238,831	57.17	140	ST14	KL2	Urine	4	>32	4
**CRE-212**	472,776,246	2,073,510	115	5,807,665	203,922	56.98	81	ST258		Other	1	2	1
**CRE-213**	845,167,838	5,597,138	458	5,736,292	149,643	57.1	147	ST512	KL107	Respiratory Tract	1	0.25	16
**CRE-214**	557,032,960	3,688,960	459	5,791,172	198,569	57.12	96	ST14	KL2	Urine	8	>32	>32
**CRE-216**	1,263,312,206	8,366,306	438	5,784,943	134,034	57.09	218	ST258		Urine	4	16	16
**CRE-219**	792,987,674	5,251,574	530	5,828,789	224,396	57.1	136	ST14	KL2	Respiratory Tract	4	>32	4
**CRE-220**	721,568,600	4,778,600	268	5,734,329	130,607	57.13	126	ST258	KL107	Respiratory Tract	4	8	8
**CRE-221**	713,994,440	4,728,440	273	5,824,897	135,336	57.03	123	ST258	KL107	Urine	8	>32	16
**CRE-225**	1,135,528,154	7,520,054	309	5,818,598	139,625	57.05	195	ST258	KL107	Blood	>32	>32	>32
**CRE-226**	1,051,276,194	6,962,094	342	5,940,219	127,177	56.92	177	ST258	KL107	Other	16	>32	>32
**CRE-227**	878,957,410	5,820,910	172	6,080,403	122,482	56.53	145	ST258	KL107	Respiratory Tract	>32	>32	>32
**CRE-228**	775,741,662	5,137,362	253	5,736,107	137,353	57.14	135	ST258	KL107	Urine	16	>32	>32
**CRE-229**	574,616,910	3,805,410	261	6,170,814	115,770	56.46	93	ST258	KL107	Urine	4	16	4
**CRE-230**	857,771,204	5,680,604	367	5,735,956	222,110	57.15	150	ST14	KL2	Urine	1	2	2
**CRE-231**	944,829,650	6,257,150	229	6,090,213	126,390	56.51	155	ST258	KL107	Urine	>32	>32	>32
**CRE-232**	1,612,049,726	10,675,826	430	5,884,526	138,031	57.02	274	ST258	KL107	Respiratory Tract	16	>32	>32
**CRE-233**	808,315,080	5,353,080	432	5,640,746	357,161	56.92	143	ST231		Urine	>32	>32	>32
**CRE-234**	1,275,964,798	8,450,098	292	5,933,164	137,640	56.83	215	ST258		Urine	2	8	4
**CRE-235**	699,999,458	4,635,758	165	5,917,094	172,931	56.57	118	ST147		Respiratory Tract	>32	>32	>32
**CRE-236**	580,365,782	3,843,482	179	5,473,066	203,894	57.37	106	ST37	KL38	Urine	2	1	4
**CRE-237**	605,382,254	4,009,154	204	5,672,780	154,281	57.06	107	ST258	KL107	Blood	4	>32	>32
**CRE-239**	1,115,197,212	7,385,412	306	5,928,948	157,591	56.71	188	ST258	KL107	Urine	8	16	4
**CRE-240**	931,840,026	6,171,126	401	5,992,484	137,534	56.8	156	ST258	KL107	Urine	16	>32	>32
**CRE-241**	666,475,948	4,413,748	651	5,900,177	201,269	56.89	113	ST14	KL2	Other	2	8	2
**CRE-243**	653,315,996	4,326,596	504	5,763,382	195,940	56.96	113	ST37	KL118	Other	8	>32	>32
**CRE-244**	801,369,986	5,307,086	211	6,161,396	127,177	56.47	130	ST258	KL107	Respiratory Tract	>32	>32	>32
**CRE-245**	734,778,986	4,866,086	449	5,888,035	129,666	57.03	125	ST258	KL107	Blood	8	16	16
**CRE-246**	830,930,350	5,502,850	421	5,893,002	127,179	57	141	ST258	KL107	Urine	8	>32	>32
**CRE-247**	1,009,929,072	6,688,272	342	5,822,856	126,391	57	173	ST258	KL107	Other	8	8	1
**CRE-248**	1,226,918,488	8,125,288	384	5,783,637	137,620	57.04	212	ST258	KL107	Urine	2	16	2
**CRE-249**	680,328,084	4,505,484	220	5,564,648	124,389	57.04	122	ST15	KL112	Urine	0.25	0.5	4
**CRE-251**	749,978,042	4,966,742	451	5,919,776	107,095	56.96	127	ST258	KL107	Urine	2	8	2
**CRE-253**	729,905,914	4,833,814	257	5,899,439	134,034	56.94	124	ST258	KL107	Urine	8	>32	>32
**CRE-255**	473,230,376	3,133,976	161	5,772,839	255,959	57.11	82	ST16		Blood	1	4	2
**CRE-256**	753,528,052	4,990,252	315	5,742,523	127,179	57.01	131	ST258	KL107	Other	>32	>32	>32
**CRE-257**	754,011,252	4,993,452	383	5,641,076	274,093	57.13	134	ST16		Urine	1	2	0.5
**CRE-258**	395,655,032	2,620,232	274	5,808,374	134,034	57.06	68	ST258	KL107	Urine	2	4	8
**CRE-259**	680,086,484	4,503,884	309	5,935,607	143,815	56.8	115	ST258		Urine	4	16	4
**CRE-260**	574,415,778	3,804,078	213	5,712,447	198,353	57.15	101	ST14	KL2	Respiratory Tract	2	8	2
**CRE-261**	416,637,690	2,759,190	302	5,713,573	185,837	57.14	73	ST14	KL2	Blood	4	16	2
**CRE-262**	591,180,402	3,915,102	288	5,498,384	125,025	57.29	108	ST15	KL112	Urine	4	2	2
**CRE-264**	1,848,758,836	12,243,436	339	5,781,167	135,840	57.12	320	ST258	KL107	Other	2	>32	2
**CRE-265**	416,462,708	1,785,828	129	5,621,785	267,080	57.2	74	ST14	KL2	Other	1	4	2
**CRE-266**	802,158,810	5,312,310	304	6,143,301	127,781	56.49	131	ST258	KL107	Other	>32	>32	>32
