# Supplementary figures and images for: Genomic Features Associated with the Degree of Phenotypic Resistance to Carbapenems in Carbapenem-Resistant Klebsiella pneumoniae
Source: mSystems. 2021 Sep 14;6(5):e00194-21. doi: 10.1128/mSystems.00194-21 (PMC8547452; doi:10.1128/mSystems.00194-21)

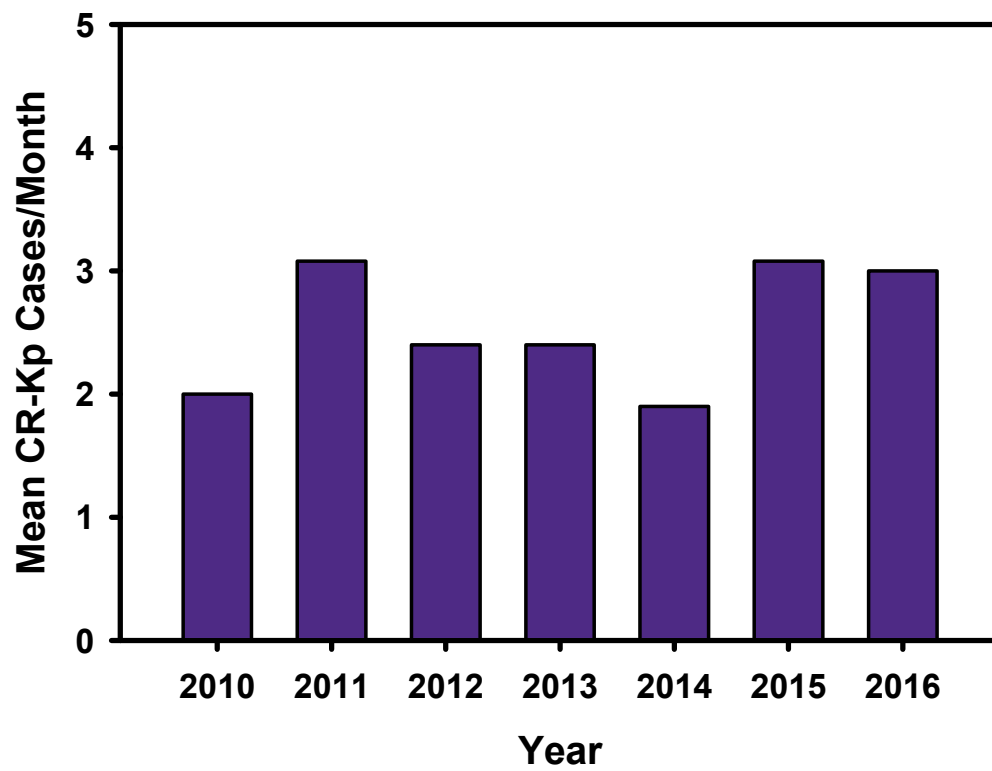

Supplement: FIG S1 [file msystems.00194-21-sf001.pdf]

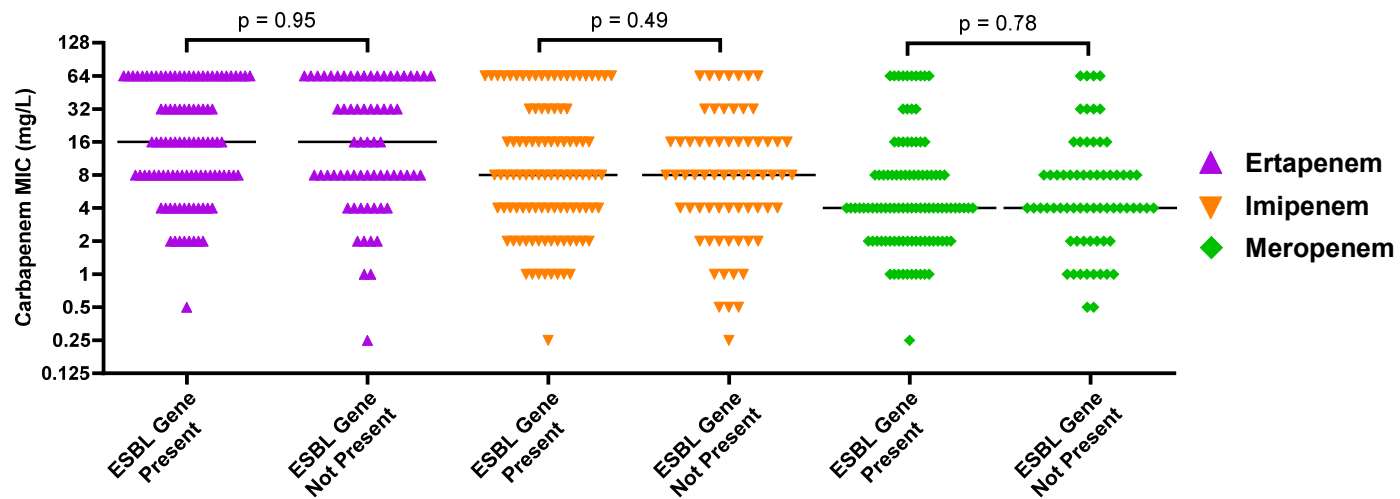

Supplement: FIG S2 [file msystems.00194-21-sf002.pdf]

A. ESBL Gene Presence and OmpK35 Function

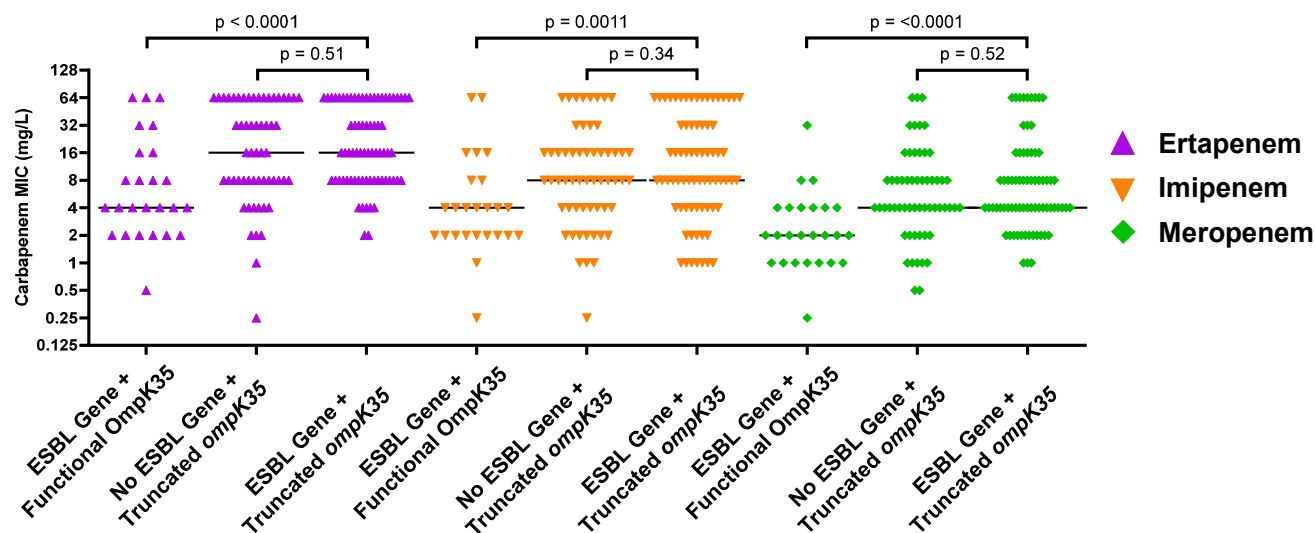

B. ESBL Gene Presence and OmpK36 Function

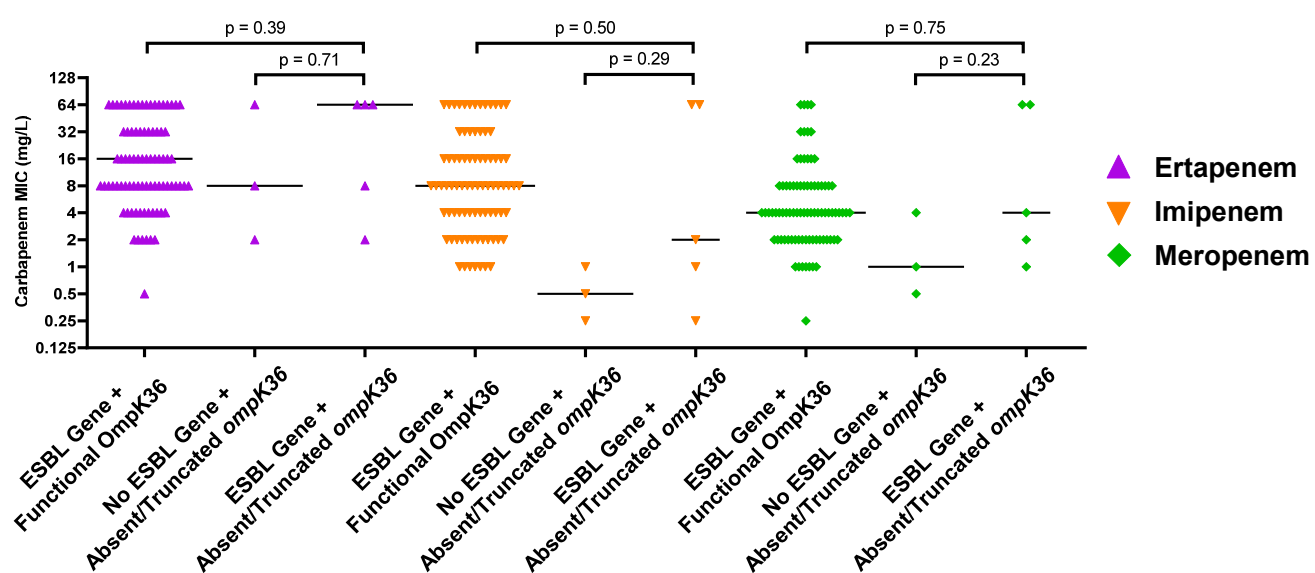

Supplement: FIG S3 [file msystems.00194-21-sf003.pdf]

A.

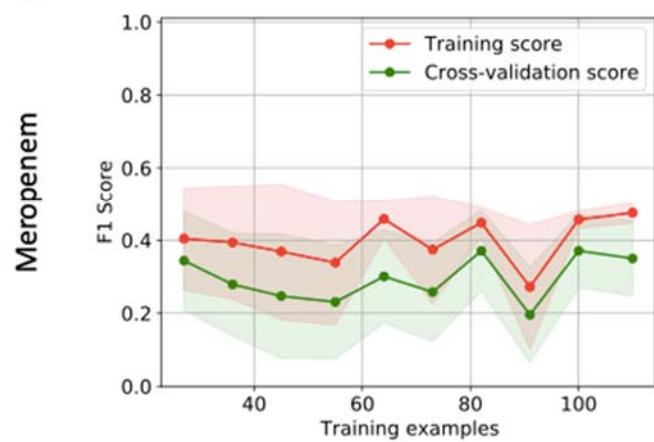

B.

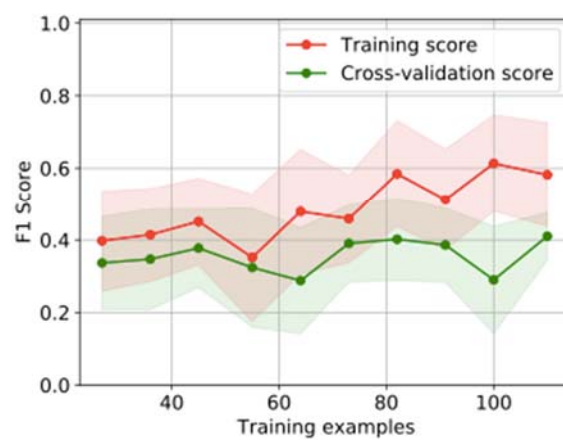

C.

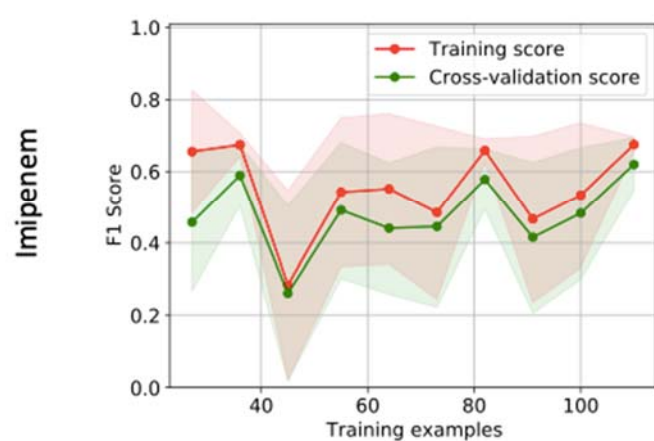

D.

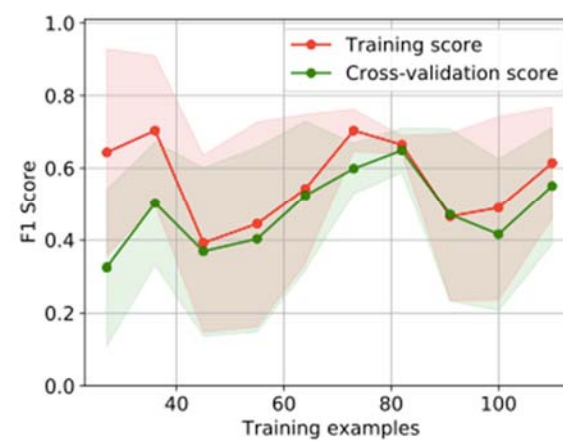

Supplement: FIG S4 [file msystems.00194-21-sf004.pdf]
